# HCR-Proxy resolves site-specific proximal RNA microenvironments at subcompartmental resolution

**DOI:** 10.1093/nar/gkag086

**Published:** 2026-02-23

**Authors:** Anja Trupej, Valter Bergant, Jona Novljan, Martin Dodel, Tajda Klobučar, Maksimiljan Adamek, Flora C Y Lee, Karen Yap, Eugene Makeyev, Boštjan Kokot, Luka Čehovin Zajc, Andreas Pichlmair, Iztok Urbančič, Faraz K Mardakheh, Miha Modic

**Affiliations:** National Institute of Chemistry, Ljubljana, SIovenia; Institute of Biological and Chemical Systems, Karlsruhe Institute of Technology, Germany; Center for Synthetic Genomics (SynGen) Heidelberg-Karlsruhe-Mainz, Germany; PhD Program ‘Biosciences’, Biotechnical Faculty, University of Ljubljana, Ljubljana, SIovenia; National Institute of Chemistry, Ljubljana, SIovenia; Institute of Virology, School of Medicine and Health, Technical University Munich, Munich, Germany; National Institute of Chemistry, Ljubljana, SIovenia; Institute of Biological and Chemical Systems, Karlsruhe Institute of Technology, Germany; Center for Synthetic Genomics (SynGen) Heidelberg-Karlsruhe-Mainz, Germany; Department of Genomics and Developmental Biology, Zoological Institute, Karlsruhe Institute of Technology, Germany; Department of Biochemistry, University of Oxford, United Kingdom; National Institute of Chemistry, Ljubljana, SIovenia; Institute of Biological and Chemical Systems, Karlsruhe Institute of Technology, Germany; Center for Synthetic Genomics (SynGen) Heidelberg-Karlsruhe-Mainz, Germany; PhD Program ‘Biosciences’, Biotechnical Faculty, University of Ljubljana, Ljubljana, SIovenia; National Institute of Chemistry, Ljubljana, SIovenia; Institute of Biological and Chemical Systems, Karlsruhe Institute of Technology, Germany; Center for Synthetic Genomics (SynGen) Heidelberg-Karlsruhe-Mainz, Germany; PhD Program ‘Biosciences’, Biotechnical Faculty, University of Ljubljana, Ljubljana, SIovenia; Centre for Developmental Neurobiology, King’s College London, London, United Kingdom; Centre for Developmental Neurobiology, King’s College London, London, United Kingdom; Centre for Developmental Neurobiology, King’s College London, London, United Kingdom; Jozef Stefan Institute, Ljubljana, SIovenia; Faculty of Computer and Information Science, University of Ljubljana, Ljubljana, SIovenia; Institute of Virology, School of Medicine and Health, Technical University Munich, Munich, Germany; Institute of Virology, Helmholtz Center Munich, Munich, Germany; German Centre for Infection Research (DZIF), Partner site Munich, Germany; Jozef Stefan Institute, Ljubljana, SIovenia; Department of Biochemistry, University of Oxford, United Kingdom; National Institute of Chemistry, Ljubljana, SIovenia; Institute of Biological and Chemical Systems, Karlsruhe Institute of Technology, Germany; Center for Synthetic Genomics (SynGen) Heidelberg-Karlsruhe-Mainz, Germany; Department of Genomics and Developmental Biology, Zoological Institute, Karlsruhe Institute of Technology, Germany; Department of Basic and Clinical Neuroscience, Institute of Psychiatry, Psychology and Neuroscience, King’s College London, United Kingdom

## Abstract

The spatial organization of RNA-scaffolded condensates is fundamental for understanding of basic cellular functions, but may also provide pivotal insights into diseases. One of the major challenges to understanding the role of condensates is the lack of technologies to map condensate-scale protein architecture at subcompartmental resolution. To address this, we introduce HCR-Proxy, a proximity labelling technique that couples hybridization chain reaction (HCR)-based signal amplification with *in situ* proximity biotinylation (Proxy), enabling proteomic profiling of RNA-proximal proteomes at subcompartmental resolution. We applied HCR-Proxy to nascent pre-rRNA targets to investigate the distinct proteomic signatures of the nucleolar subcompartments and to uncover a spatial logic of protein partitioning shaped by RNA sequence. Our results demonstrate the ability of HCR-Proxy to provide spatially resolved maps of RNA interactomes within the nucleolus, offering new insights into the molecular organization and compartmentalization of condensates. This subcompartment-specific nucleolar proteome profiling enabled integration with deep learning frameworks, which effectively confirmed a sequence-encoded basis for protein partitioning across nested condensate subcompartments, characterized by antagonistic gradients in charge, molecular weight, and RNA-binding domains. HCR-Proxy thus provides a scalable platform for spatially resolved RNA interactome discovery, bridging transcript localization with proteomic context in native cellular environments.

## Graphical Abstract

**Graphical Abstract:**
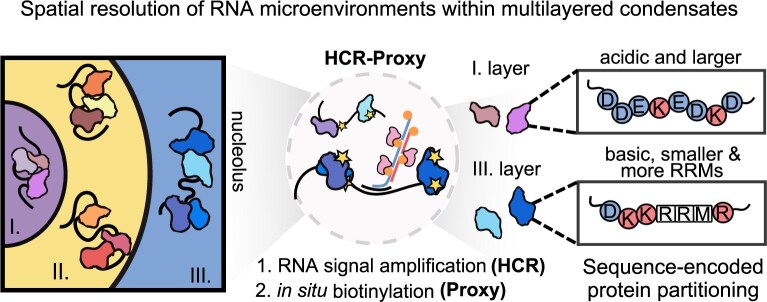

## Introduction

The cellular milieu is organized into a myriad of membraneless compartments or biomolecular condensates, many of which are formed through multivalency-driven phase separation [1]. RNA molecules often serve as a nucleation seed, providing an architectural blueprint for the recruitment of RNA-binding proteins [2] and ultimately condensate formation. Many condensates are heterogeneous entities, displaying various degrees of internal subcompartmentalization. Nucleoli [3], Cajal bodies [4], paraspeckles [5], and nuclear speckles [6] are among the best known examples of macro-assemblies, with information workflow organized in nested-like structures [4]. Therefore, characterizing molecular assemblies on a subcompartment-wide scale is of vital importance for understanding the flux of biomolecules governed by the spatial organization and, most importantly, its contribution to the cell’s homeostasis.

High-resolution microscopy using immunofluorescence (IF) is widely employed to study condensate composition [7–9], while RNA–protein interaction mapping has relied on affinity-based approaches [10–12], and newer RNA-targeted degradation methods [13, 14]. Although these techniques identify direct interactors, they lack spatial context information crucial for compartment-level analysis. Proximity labelling (PL) overcomes this caveat by utilizing an engineered biotinylating enzyme (e.g. APEX2 [15, 16], HRP [17], or TurboID [18]), co-localizing with the molecule of interest. Upon addition of a biotin substrate, enzymes catalyse short-lived reactive species which are covalently deposited on biomolecules located in close proximity to enzymes. Subsequently, biotinylated molecules are enriched by streptavidin pull-down and identified by mass spectrometry (MS) or nucleic acid sequencing [15]. Despite a broad repertoire of *in vivo* PL methods [19–23], such approaches require genetic manipulations of the cells and exhibit a low signal-to-noise ratio and experimental reproducibility. To overcome these limitations, *in situ* PL approaches [24–26] have been developed that use labelled antisense oligos to biotinylate targeted RNA neighbourhoods without genetic editing. Despite bypassing the need for genetic manipulations, challenges remain in labelling specificity, spatial resolution, and inefficient biotinylation, thereby preventing the characterization of the proximal interactome at subcompartmental scale. Thus, there is a pressing need to develop a biotinylation method with enhanced labelling and spatial resolution capacity to map spatially confined RNA interactomes at subcompartmental resolution.

To address this need, we have developed HCR-Proxy, an improved proximity labelling approach to identify site-specific RNA proteomes with subcompartment spatial resolution. HCR-Proxy is a hybrid method composed of a signal amplification [hybridization chain reaction (HCR)] and an *in situ* biotinylation step (Proxy). Unlike current *in situ* PL methods, where biotinylation efficiency depends on the number of labelled probes recognized by the PL enzymes, HCR-Proxy uses metastable hairpins to amplify the biotinylation signal. This results in probe-independent RNA labelling that is suitable for studying proximal proteomes of challenging RNA molecules with limited sequence space for antisense probe design. To improve the resolution of the labelling, we concomitantly applied automated stimulated emission depletion (STED) microscopy and profiled various molecular crowding conditions to technically refine the method. We performed HCR-Proxy against different nuclear RNA targets of different abundances, and demonstrated its flexibility and superior biotinylation efficiency. As a proof of concept, we applied HCR-Proxy to target specific nucleolar biogenesis-driven rRNA transcripts, demonstrating the method’s ability to specifically enrich distinct RNA-proximal proteomes within a nested condensate. Furthermore, the integration of deep learning models showed that basic protein features such as molecular weight, composition, and net charge can classify proteins located in different RNA-scaffolded neighbourhoods. Overall, this demonstrates that HCR-Proxy is a powerful tool with unprecedented spatial resolution for deciphering higher-order interactomes within spatially restricted RNA-scaffolded condensates. Thereby HCR-Proxy expands the toolbox for spatial proteomics by unifying RNA programmability with enzymatic labelling and biophysical modulation.

## Materials and methods

### Cell culture

Low-passage, wild-type (WT) mouse pluripotent stem cells IDG3.2 PSCs (129S8/B6 background [27]) and endogenously tagged Polr1A-GFP mouse embryonic stem cells (mESCs) were cultured in a humidified incubator at 37°C, 5% CO_2_, feeder-free, on cell culture dishes (TPP) coated with 0.1% gelatin (Millipore, cat #ES-006-B). Cells were maintained in N2B27 medium composed of 1:1 Neurobasal medium (Thermo Fisher Scientific, cat #21 103 049) and Dulbecco’s modified Eagle’s medium (DMEM)-F12 (Thermo Fisher Scientific, cat #11 320 074), containing N2 (Thermo Fisher Scientific, cat #17 502 001) and B27 (Thermo Fisher Scientific, cat #17 504 001) supplements, 12 ng ml^−1^ leukaemia inhibitory factor (LIF) (Qkine, cat #104 278), with additional use of small molecule inhibitors: for the condition commonly termed 2iLIF, 1 mM MEK inhibitor PD0325901 (Axon Medchem, cat #1408) and 3 mM GSK3 inhibitor CHIR99021 (Sigma, cat #SML1046) were used. Cells were passaged every 2–3 days using Accutase (Sigma, cat #A6964).

### Generation of CRISPR/Cas9 genome engineering mESC lines

For the generation of Polr1A–green fluorescent protein (GFP), specific guide RNAs (gRNAs) targeting upstream of the stop codon (gRNA sequence AGACAATGCTGCTATCTTAG) were cloned into a modified version of the SpCas9-T2A-GFP/gRNA plasmid (px458172, Addgene plasmid #48 138), where we fused a truncated form of human Geminin (hGem) to SpCas9 in order to increase homology-directed repair efficiency, generating SpCas9-hGem-T2A-GFP/gRNA. To generate Polr1A–GFP targeting donors, double-stranded DNA (dsDNA) gene fragments were synthesized and cloned into a vector carrying ∼400 bp homology arms (from IDT). For targeting, WT ESCs were transfected with a 4:1 ratio of donor oligonucleotide and SpCas9-hGem-T2A-GFP/gRNA construct. Positively transfected cells were isolated based on GFP expression using fluorescence-activated cell sorting (FACS) and plated at clonal density in ESC medium 2 days after transfection. After 5–6 days, single colonies were picked and plated on 96-well plates. These plates were then duplicated 2 days later and individual clones were screened for the desired mutation by PCR. Cell lysis in 96-well plates, PCR on lysates, and restriction digests were performed. The presence of the desired Polr1A–GFP insertions in putative clones was confirmed by Sanger sequencing.

Recombinant proximity labelling enzymes

### Molecular cloning

Plasmid pML433 [28] for bacterial production of fusion protein APEX2–DIG with an N-terminal T7 tag, a flexible linker between APEX2 and DIG, the C-terminal TEV-cleavage site, and a hexahistidine tag was a kind gift from Dr Karen Yap, King’s College London, United Kingdom. In this plasmid, APEX2 is inserted via EcoRI and HindIII restriction sites, which were used to replace APEX2 with TurboID. The gene fragment for TurboID was taken from Addgene Plasmid #107 177 and purchased from IDT with 5′ EcoRI and 3′ HindIII restriction sites. The TurboID gene fragment and pML433 with APEX–DIG were double digested with FastDigest EcoRI (cat #FD0274) and FastDigest HindIII (cat #FD0505) (Thermo Fisher Scientific). The restriction reactions were resolved by agarose gel electrophoresis (AGE; 1% agarose gel, TBE buffer, 30 min at constant 110 V). pML433 without APEX2 and the digested TurboID gene fragment were isolated from the gel with the use of the Zymoclean Gel DNA Recovery Kit (Zymo Research) and further purified with the use of the Zymoclean DNA Clean & Concentrator-25 Kit (Zymo Research). T4 DNA ligase (Thermo Fisher Scientific, cat #EL0011) was then used to ligate TurboID into pML433 in an overnight reaction performed at 16°C. The ligation mixture was transformed into chemically competent *Escherichia coli* cloning strain DH5ɑ via the standard heat shock procedure. Bacteria were plated on an LB agar plate supplemented with 30 μg ml^−1^ kanamycin (LBK-agar). After overnight incubation at 37°C, colonies were analysed via colony PCR with the use of DreamTaq PCR Master Mix (Thermo Fisher Scientific, cat #K1081) and T7-promoter and T7-terminator annealing oligonucleotides (purchased from IDT). Positive clones were transferred into liquid LB medium supplemented with 30 μg ml^−1^ kanamycin (LBK) and grown overnight (37°C and shaking at 150 rpm). The following day, plasmid DNA was isolated with the use of the ZR Plasmid Miniprep-Classic Kit (Zymo Research, cat #D4015). Isolated DNA was sent for Sanger sequencing (Macrogen). The clone with the correct sequence and best *A*_260_/*A*_280_ and *A*_260_/*A*_230_ ratios (as measured by NanoDrop One, Thermo Fisher Scientific) was chosen for subsequent work.

### Bacterial culture

Plasmid pML433 with the construct T7-tag_APEX2_linker_DIG_hexahistidine-tag (APEX2-DIG) or T7-tag_TurboID_linker_DIG_hexahistidine-tag (TurboID-DIG) was transformed into chemically competent *E. coli* expression strain BL21(DE3) via the standard heat shock procedure. Bacteria were plated on an LB agar plate supplemented with 30 μg ml^−1^ kanamycin. After overnight incubation at 37°C, a single bacterial colony was transferred to 10 ml of LB medium containing 30 μg ml^−1^ kanamycin. The culture was incubated with shaking at 37°C and 150 rpm for 8 h. Then, 100 µl of starter culture was transferred to 50 ml of fresh LBK. The culture was left overnight with shaking at 37°C and 150 rpm. The next day, 2.5 ml of overnight culture was added to 250 ml of TB medium supplemented with 100 mM phosphate buffer (pH 7.0) and 30 μg ml^−1^ kanamycin (TBK). For the APEX2–DIG construct, the medium was also supplemented with 0.1 mM FeSO_4_. The cultures were grown with shaking at 37°C and 150 rpm to an OD_600_ of 1.0. The cultures were briefly cooled on ice, induced with 0.5 mM isopropyl-β-d-thiogalactopyranoside (IPTG), and left overnight (∼18 h) with shaking at 20°C and 150 rpm. The next day, cultures were centrifuged, the supernatant was discarded, and the resulting bacterial cell pellets were frozen at −80°C until use.

### Protein purification

Protein purification was done following a slightly modified protocol established by Yap and colleagues [28]. Briefly, the bacterial cell pellet (5 g for the APEX2 construct and 3 g for the TurboID construct) was left to thaw on ice. Then, ice-cold lysis buffer [50 mM HEPES (pH 8.0), 200 mM NaCl, 10 mM MgCl_2_, 10% glycerol, 2.5 mM 2-mercaptoethanol, 1 mM PMSF, 0.1% (v/v) Triton X-100, 250 U of benzonase, 0.5 mg ml^−1^ lysozyme] was added (pellet:buffer w/v ratio = 1:10) and the pellet was gently resuspended. The cells were lysed by sonication on an ice bath (Cole Parmer Ultrasonic Processor; 38% amplitude, 1 s pulse time, 2 s pause, total pulse time 5 min). The lysed cells were centrifuged at 50 000 *g* for 30 min at 4°C. The supernatant was filtered through a 0.22 µm filter. Protein purification was performed on an ÄKTA pure M25 chromatography system (Cytiva), which was kept in a refrigerator at 4°C. The clarified lysate was loaded onto a HR 10/20 Ni-NTA Superflow (Qiagen) column, which was pre-equilibrated with IMAC buffer A [25 mM HEPES (pH 8.0), 200 mM NaCl, 5% glycerol, 2.5 mM 2-mercaptoethanol]. The column was washed with stepwise increasing concentrations of IMAC buffer B [25 mM HEPES (pH 8.0), 200 mM NaCl, 5% glycerol, 2.5 mM 2-mercaptoethanol, 500 mM imidazole], 2.5% IMAC buffer B [10 column volumes (CVs)], and 5% IMAC buffer B (10 CVs). Elution was carried out with 50% IMAC buffer B. The protein peak from IMAC was loaded onto a HiLoad Superdex 200 PG 16/60 column (GE Healthcare), which was pre-equilibrated with SEC column buffer [25 mM HEPES (pH 8.0), 200 mM NaCl, 5% glycerol, 1 mM DTT]. The chosen fractions were analysed by SDS PAGE (mPAGE 4–12% Bis-Tris pre-cast gels, Merck Millipore, cat #MP41G10) and fractions containing high amounts of purified protein were pooled together. Protein concentration was estimated by NanoDrop One. The APEX2–DIG construct was stored as is (concentration 2 mg ml^−1^), while the TurboID–DIG construct was concentrated with the use of a Pierce Protein Concentrators PES, 10K molecular weigh cut-off (Thermo Fisher Scientific), to a final concentration of 1 mg ml^−1^. Proteins were aliquoted and stored at −80°C.

Semi-quantitative tests for APEX2–DIG peroxidase activity and its DIG binding ability were performed by the protocol previously described [25]. TurboID–DIG biotinylation activity was determined by HCR-Proxy IF staining.

### DNA probes and DIG-labelled hairpins for proximity labelling

DNA oligonucleotide split probes (Supplementary Table S1) complementary to precursor ribosomal RNA (A′ UP), processed pre-rRNA or A′ DOWN (A′ −A0), intermediate ribosomal RNA (ITS2), *Malat1, Norad*, and *Efl1* were designed by a custom software (ÖzpolatLab-HCR, 2021 - Github [29]). Sequences generated by the software were purchased from IDT, resuspended in 100 µM nuclease-free water, and mixed for each probe stock with a final 5 µM concentration. DIG and fluorophore-labelled HCR hairpins (Supplementary Table S1) were custom designed by Molecular Instruments Inc. and stored in a light-tight container at −20°C.

### Immunofluorescence and HCR-FISH

HCR-fluorescence *in situ* hybridization (FISH) was performed according to the published protocol [30]. WT IDG3.2 mESCs were plated on a Geltrex™ hESC-Qualified (Thermo Fisher Scientific, cat #A1569601) coated 8-well glass-bottom IBIDI plate 1 day prior to fixation. Cells were fixed with 0.5 mg ml^−1^ dithiobis(succinimidyl propionate) (DSP; Thermo Fisher Scientific, cat #22 585) in 1× PBS for 40 min at room temperature, washed three times with 1× PBS and 20 mM Tris–HCl, pH 7.5, for 5 min each wash, and permeabilized with 70% ethanol overnight at 4°C. Hybridization and amplification steps were performed as described in the protocol. For IF staining, after amplification, samples were thoroughly washed three times with 5× SSCT [5× SSC (Invitrogen, cat #15 557 044), 0.1% Tween-20], for 5 min each wash, washed twice with 1× PBS, and blocked with IF blocking buffer [0.3% Triton X-100 + 3% BSA + 10 U ml^−1^ Rnasin (Promega, cat# N2615)] for 1 h at room temperature. To visualize RNA labelled with DIG, we used DyLight® 594 anti-DIG antibody (goat, Vectorlabs, cat# DI-7594-.5, 1:100 dilution). To validate co-localization with nuclear condensates and to confirm localization of candidate proteins, we used the following antibodies against: SC-35 (mouse, Santa Cruz Biotechnology, cat #sc-53518, 1:200 dilution), NPM1 (mouse, ThermoFisher Scientific, cat #32-5200, 1:100 dilution), FBL (rabbit, Abcam, cat #ab5821, 1:200 dilution), TXNRD1 (rabbit, Proteintech, cat #11117-1-AP, 1:200 dilution), and GFP (rabbit, ThermoFisher Scientific, cat #A-21311, 1:100 dilution). Samples with antibodies were incubated with 0.8% BSA in 4× SSC (Blocking buffer) supplemented with 10 U ml^−1^ Rnasin inhibitors overnight at 4°C. The next day, samples were washed with 4× SSC, 4× SSC, and 0.1% Triton X-100 + 4× SSC, for 10 min each wash, and incubated with secondary antibodies, anti-mouse Alexa Fluor 647 conjugated (donkey, Invitrogen, cat #A-31571, 1:400 dilution) or anti-rabbit Alexa Fluor 647 conjugated (donkey, Abcam, cat #ab150075, 1:400 dilution) for 1 h at room temperature. Afterwards, cells were washed with 4× SSC, 4× SSC, and 0.1% Triton X-100 + 4× SSC, for 10 min each wash, and cell nuclei were stained with DAPI (ThermoFisher, cat #62 248, 1:3000 dilution) in 1× PBS for 8 min at room temperature. Samples were mounted with 250 μl of Fluoromount G (Thermo Fisher Scientific, cat #00-4958-02), sealed with parafilm, and saved at 4°C until imaging.

### HCR-Proxy

For HCR-Proxy MS experiments, ~5 million cells per replicate were used in four replicates for each RNA target (bait) for subcellular HCR-Proxy-targeted interactomes, while for subnucleolar HCR-Proxy experiment ~8 million cells per replicate were used in four replicate per each RNA target.

Cells grown in 10 cm dishes (HCR-Proxy MS) or 8-well glass-bottom IBIDI coverslips (HCR-Proxy FISH/IF staining) (IBIDI) (90% confluency) were washed once with 1× PBS, aspirated, and fixed with 0.5 mg ml^−1^ DSP in 1× PBS for 40 min at room temperature. Samples were then washed three times with 1× PBS and 20 mM Tris–HCl, pH 7.5, for 5 min each wash, and permeabilized with 70% ethanol overnight at 4°C. The next day, cells were rehydrated with two washes of 2× SSC, scraped into DNA LoBind tubes (Eppendorf, cat #30 108 051) with 2× SSC supplemented with 1% BSA (pluriSelect Life Science UG, cat# 60-00020-11) and 0.5× phosSTOP (Sigma Aldrich, cat #4 906 837 001) and centrifuged for 5 min at 3400 rpm at room temperature to remove the supernatant. From this point on, all steps were carried out within DNA LoBind tubes, rotated on the rotor and after each incubation or wash, centrifuged for 5 min at 3400 rpm at room temperature to remove any residue. HCR hybridization and amplification steps were performed by following the HCR-FISH protocol [30]. Additionally, to prevent RNA degradation and other enzymatic activity, 1× phosSTOP and 10 U ml^−1^ Rnasin inhibitors were added to pre-hybridization and pre-amplification steps; 1× phosSTOP and 25 U ml^−1^ Rnasin inhibitor were supplemented to tubes for overnight incubations such as for probe hybridization and HCR (hairpin) amplification.

After HCR amplification, cells were centrifuged and washed three times with 5× SSCT for 5 min at room temperature to remove the unbound hairpins. Cells were blocked with the Blocking buffer (0.8% BSA in 4× SSC) and supplemented with 50 U ml^−1^ Rnasin inhibitor for 30 min at room temperature. Afterwards cells were incubated with 2.7 µg ml^−1^ TurboID–DIG or APEX2–DIG PL enzyme in the Blocking buffer for 1 h at room temperature on the rotor. To remove any unbound PL enzyme, cells were thoroughly washed by once with 4× SSC, twice with 4× SSC + 0.1% Triton and twice with 4× SSC for 10 min each. Next, cells were transferred to 2 ml of DNA LoBind tubes (Eppendorf, cat# 30 108 078) and incubated in 0.5 ml of Labeling solution [2.5% BSA and 20% trehalose (AMSBIO EUROPE B.V., cat #AMS.TS1M-100)] for 5 min on the rotor. PL was then performed by the addition of an equal volume of ice-cold Labelling solution supplemented with 1 mM biotin–phenol and 0.2 mM hydrogen peroxide (Merck, cat #H1009-5ML) and gentle rotation on ice for a defined amount of time. To stop the biotinylation reaction, cells were quenched three times with 1 ml of ice-cold Quencher solution [10 mM sodium ascorbate (Sigma, cat #SI-A4034-100G) and 5 mM Trolox (Sigma, cat #AL-238813-1G) in 1× PBS] and thoroughly shaken. The last wash was performed in 1.5 ml DNA LoBind tubes with an additional final centrifugation step to remove any remaining Quencher solution. Samples labelled in tubes were then analysed by immunoblotting and MS; coverslips were used for HCR-Proxy FISH/IF imaging.

To evaluate the effect of crowding agents on the biotinylation radius, eight different Labelling solutions were prepared: 2.5% BSA, 5% BSA, 40% trehalose, 2.5% BSA and 20% trehalose, 1.25% BSA and 30% trehalose, 3% PEG8000 (New England Biolabs, cat #B1004S), 15% PEG8000, and 1 M sucrose (Sigma, cat #16 104). All solutions were prepared in 1× PBS, and 1× PBS served as a biotinylation positive control.

### Isolation of biotinylated proteins

Biotinylated cells were lysed within tubes with 0.3 ml of High SDS RIPA lysis buffer (150 mM NaCl, 1 mM EDTA, pH 8.0, 50 mM Tris–HCl, pH 8.0, 1% NP-40, 0.5% sodium deoxycholate, 0.5% SDS) supplemented with 1× phosSTOP + 1× cOmplete EDTA-free protease inhibitor (Sigma, cat #SRO-5056489001) + 10 mM sodium ascorbate + 5 mM Trolox + 50 mM DTT (ZellBio, cat #DTT25), transferred into Bioruptor tubes (Diagenode, cat #C30010016), and incubated on ice for 15–20 min while suspended on a shaker. Samples were sonicated using a Bioruptor Pico system (Diagenode) with a built-in cooling system (Bioruptor® Cooler), for 15 cycles of 30 s ON/30 s OFF at the low-frequency setting. Lysates were then transferred to new DNA LoBind tubes, topped up to 1 ml with High SDS RIPA lysis buffer supplemented with 1× phosSTOP + 1× cOmplete EDTA-free protease inhibitor + 10 mM sodium ascorbate + 5 mM Trolox + 50 mM DTT, and reverse cross-linked on a thermoblock at 37°C for 60 min with shaking at 1500 rpm . Each sample was additionally supplemented with 2 µl of Turbo DNase (2 U μl^−1^, Invitrogen, cat #AM2238) and 1 µl of benzonase (≥ 250 U µl^−1^, Merck, cat #101 654). For thorough DNA denaturation and cell homogenization, we pushed the cell lysate through a 27G syringe (8–10 times), followed by boiling the lysate for 5 min at 95°C and centrifugation at 15 000 *g* for 10 min at 4°C. Clear supernatant was transferred to a new tube and stored at −70°C until needed.

Before streptavidin pull-down, samples were diluted with an additional 0.5 ml of High-SDS RIPA lysis buffer supplemented with 1× phosSTOP and pre-cleared with Dynabeads Protein G (Invitrogen, cat #10004D). For pre-clearing, 15 µl of Dynabeads per sample were washed three times with iCLIP lysis buffer (50 mM Tris–HCl, pH 7.4, 100 mM NaCl, 1% Igepal CA-630, 0.1% SDS, 0.5% sodium deoxycholate), then resuspended with reverse cross-linked lysates and incubated for 45 min at 4 ⁰C with rotation. Cleared lysates were collected using a magnetic rack and then incubated overnight at 4°C with rotation with 40 µl of Pierce streptavidin magnetic beads per sample (Thermo Fisher Scientific, cat# 88 817), pre-washed three times with iCLIP lysis buffer. The following day, beads were pelleted and subjected to sequential washes: three times with an iCLIP lysis buffer supplemented with 0.5% SDS; twice with High salt washing buffer (50 mM Tris–HCl pH 7.4, 1 M NaCl, 1 mM EDTA, 1% Igepal CA-630, 0.1% SDS, 0.5% sodium deoxycholate); once with 2 M urea in 10 mM Tris–HCl, pH 8.0 for 1 min; and twice with iCLIP lysis buffer (0.5% SDS). For the final wash, beads were transferred to a new tube and washed five times with Wash buffer (50 mM Tris–HCl pH 7.4, 5% glycerol, 1.5 mM MgCl_2_, 100 mM NaCl) to remove any residual SDS. After the last wash, beads were transferred to a new tube and resuspended in 37 µl of the Wash buffer. A 5 µl aliquot was taken for immunoblotting and the rest of the beads were put on the magnetic rack to remove any residual buffer and then stored at −70°C until MS sample preparation.

### MS sample preparation and data acquisition

Protein-loaded beads underwent on-bead digestion, where they were resuspended in 50 μl of 8 M urea (Merck, cat #U1250) and 50 mM ammonium bicarbonate (Merck, cat #A6141) (ABC buffer). Reduction was performed by adding DTT to a final concentration of 10 mM followed by incubation at 25°C with agitation of 1200 rpm. After 30 min, samples were alkylated by adding iodoacetamide (VWR, cat #786-228) to a final concentration of 55 mM and incubated in the dark at 25°C with agitation of 1200 rpm for another 30 min. Next, 50 mM ABC buffer was added to each sample to a final volume of 200 μl and a urea concentration of 2 M. Overnight tryptic digestion was then performed by adding 0.5 μg of trypsin per sample (Merck, cat #T6567) and incubation at 25°C, 1200 rpm. The next day, the supernatant was separated from the beads using a magnetic tube rack. After mixing the supernatant with 4% acetonitrile (J.T.Baker, cat #9012) and 1% TFA (Thermo Fisher, cat #85 183) (STOP4 buffer) at a ratio of 1:1, samples were desalted using the Stage Tip procedure [31] and recovered in 0.1% TFA, 0.5% acetic acid (Honeywell, cat# 33 209), 2% acetonitrile (A* buffer) for MS analysis.

Alternatively, beads were processed using filter-aided sample preparation (FASP), whereby they were first resuspended in 40 μl of 4% SDS (Merck, cat #75 746), 100 mM Tris–HCl pH 7.5, and incubated at 95°C for 5 min before the supernatant was separated and processed according to the FASP protocol [32]. The resulting peptides were desalted using the Stage Tip procedure [31] and recovered in 0.1% TFA, 0.5% acetic acid, 2% acetonitrile (A* buffer) for MS analysis.

LC-MS analysis was performed on a Q Exactive-plus Orbitrap mass spectrometer coupled with a nanoflow ultimate 3000 RSL nano HPLC platform (Thermo Fisher Scientific), using a 50 cm × 75 μm RSLC C18 column (Thermo Fisher Scientific) and a 123 min gradient of 3–35% of Buffer B (0.1% FA in acetonitrile) against Buffer A (0.1% FA in LC-MS gradient water). A total of 6 μl out of 7 μl were injected, and MS was carried out as previously described [33].

### Immunoblotting

A 5 µl aliquot of protein-loaded beads in Wash buffer was mixed 1:1 with 50 mM DTT and 4× NuPAGE LDS Sample Buffer (Invitrogen, cat #NP0007), and boiled for 10 min at 95°C to elute proteins. The samples were centrifuged, placed on a magnetic rack and eluates were collected and analysed by SDS–PAGE using NuPAGE® Novex 4–12% Bis-Tris gels (Thermo Fisher Scientific, cat #NP0322BOX), followed by an electrotransfer to nitrocellulose membranes using the Trans-Blot Turbo Transfer System (Bio-Rad) according to the manufacturer’s instructions. The membranes were blocked in 3% BSA and 0.1% Tween-20 for 1 h on the rotor at room temperature, then incubated for another 90 min with IRDye® 800CW Streptavidin (Li-COR, cat #926-32 230, 1:1000 dilution). Membranes were washed three times with PBS and 0.1% Tween-20, with each wash lasting 10 min, and biotinylated proteins were visualized with the iBright™ FL1500 imaging system (Thermo Fisher Scientific).

### HCR-Proxy FISH

**Figure 1.**
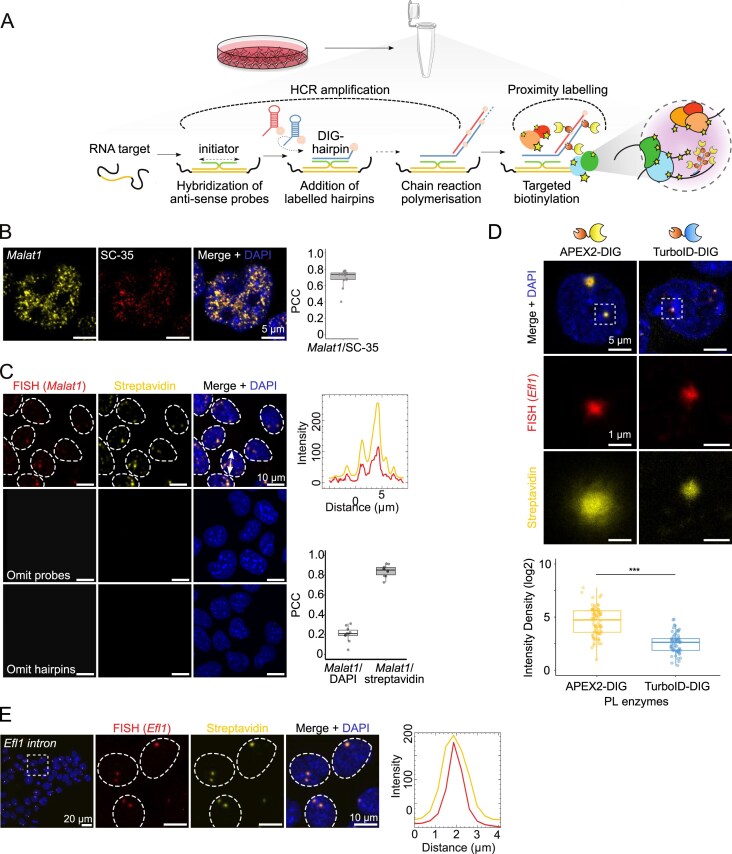
HCR-Proxy design and implementation. (**A**) Schematic overview of HCR-Proxy. The main part of the *in situ* workflow is streamlined into tubes, where chemically fixed cells are hybridized with pairs of antisense probes towards target RNA. Only the complete probe pair alignment enables signal amplification (HCR) via polymerized DIG-labelled metastable hairpins and therefore recruitment of PL enzyme to catalyse *in situ* proximity biotinylation (Proxy). (**B**) HCR-FISH IF micrographs of non-coding RNA *Malat1* (yellow, DyLight594-conjugated anti-DIG) in mESCs co-localizing with the nuclear speckle marker SC-35 (red, SC-35 antibody), merged with DAPI staining (blue). The co-localization of the signal was calculated using Pearson’s correlation coefficient (PCC) from 13 images (dots represent each cell). (**C**) HCR-Proxy IF micrographs of *Malat1* in mESCs supported with signal intensity profiles of RNA FISH (red, DyLight594-conjugated anti-DIG) and HCR-Proxy (yellow, Alexa647-conjugated streptavidin) signals. Cell nuclei were visualized with DAPI staining (blue). The co-localization of the signal was calculated using PCC from 10 images (dots represent each cell). (**D**) HCR-Proxy IF micrographs of the *Efl1* intronic transcript (red, DyLight594-conjugated anti-DIG) and its proximal vicinity labelled with two different PL enzymes, APEX2 or TurboID (yellow, Alexa647-conjugated streptavidin), merged with DAPI staining (blue). The difference in intensity density was calculated with two-sided Student’s *t*-test (***P < 0.001, **P < 0.01, *P < 0.05). (**E**) HCR-Proxy IF micrographs of the *Efl1* intronic transcript with signal intensity profiles of RNA FISH (red, DyLight594-conjugated anti-DIG) and HCR-Proxy (yellow, Alexa Fluor 647-conjugated streptavidin) signals, merged with DAPI staining (blue).

Proximity-labelled samples on 8-well IBIDI coverslips (prepared as described above) were washed twice for 5 min each with PBS and incubated overnight at 4°C with DyLight® 594 antibody (Vectorlabs, cat #DI-7594-.5, 1:100 dilution) in Blocking buffer supplemented with 10 U ml^−1^ Rnasin inhibitor. The following day, samples were washed with 4× SSC, 4× SSC + 0.1% Triton X-100, and 4× SSC, for 10 min each wash, and incubated with streptavidin STAR RED dye (Abberior, cat #STRED-0120-1MG, 1:1000 dilution) in Blocking buffer supplemented with 10 U ml^−1^ Rnasin inhibitor at room temperature for 1 h. Afterwards, cells were washed with 4× SSC, 4× SSC + 0.1% Triton X-100, and 4× SSC, for 10 min each wash, and cell nuclei were stained with DAPI (1:3000 dilution) in 1× PBS for 8 min at room temperature. Samples were mounted with 250 μl of Fluoromount G, sealed with parafilm, and saved at 4°C untill imaging. Images for Fig. 1C and E were acquired with a Leica SP5 fluorescence microscopy, while the rest of the images were acquired with STED microscopy.

### Fluorescence confocal and super-resolution imaging (STED)

Images were acquired with a customized STED microscope (Abberior instruments) using a ×60 water immersion objective. We excited fluorescently labelled RNA and streptavidin dye (biotinylation site) with pulsed lasers at 561 and 640 nm, respectively, and DAPI-stained nuclei with a CW 405 nm laser. We acquired the fluorescence intensity through a confocal pinhole set to 0.9 AU using avalanche photodiodes with 500–550, 580–625, or 655–720 nm filters (Semrock).

Automated acquisition was driven by our bespoke Python scripts generated for this manuscript [34], which ran over a grid of 10 × 10 positions for each sample on the 8-well slide. At each position, a large field-of-view image with coarse sampling was acquired in the confocal mode to identify positions with FISH signal within the nucleus (overlap with DAPI signal). For each identified nuclear condensate, an axial 3D STED cross-section image was acquired to determine the precise focus for the final 2D STED image (resolution improved laterally, *xy*). Typically, the laser powers were ~2 and 20 μW for the 640 and 561 nm laser, respectively, and 90–140 mW for the STED laser. The image size was 3–7 μm, pixel size was set to 30 nm, and pixel dwell time 10 μs. Detection was gated (gate delay 250 ps, width 8 ns), and signal was accumulated over 2–10 line repetitions to achieve a sufficient signal. For both cross-sections, a confocal image was also acquired for comparison.

In total, the automated acquisition allowed us to capture 1000 STED images of individual condensates (an average of 100 per sample) within 12 h. For each condition, ~80% of the images that met the image criteria (not recognized as an artefact) were selected for further analysis.

For confocal imaging of co-localization of TXNRD1 with A′ UP or A′ DOWN, TXNRD1 was targeted with an antibody conjugated to anti-rabbit Alexa Fluor 647 conjugated (donkey, Abcam, cat #ab150075, 1:400 dilution), while A′ UP/DOWN were labelled as described above. Imaging was performed with the same Abberior Instruments STED microscope in confocal fluorescence lifetime imaging (FLIM) mode, enabled by the Becker&Hickl SPC150 electronics. FLIM analysis was used to eliminate the cross-talk between the dyes. Namely, photon histograms in the red channel (640 nm excitation, 650–720 nm detection) were split into contributions of DyLight594 and Alexa Fluor 647 with characteristic lifetimes of 3.68 and 1.42 ns, respectively. Co-localization of the protein and RNA was assessed by calculating the spatial cross-correlation function (*G*), radially averaged for displacements (*d*) 0–5 μm (Fig. 3F), and compared with the values obtained for a randomized signal [seven different rotations and/or reflections were applied to one of the two images before cross-correlation, and the results were averaged into *G*_RAND_(*d*)]. The correlation measure values *C*(*d*) = *G*(*d*)/*G*_RAND_(*d*) > 1 indicate above-random co-localization of TXNRD1 with both A′ UP and A′ DOWN for displacements smaller than the size of the condensates, and expectedly converge to 1 (random co-localization) for larger displacements. The bands represent 80% confidence intervals across results from 10 images per condition (depicted by thin lines in the correlation line plot, or individual data points in distribution plots). The statistical tests were performed for the population of 10 images per condition, applying the one-sided Mann–Whitney test with the alternative hypothesis indicated by the symbol (e.g. *P* > for probability of A′ UP median not being greater than A′ DOWN, or *P* > 0 for the median value not being above 0).

### Image analysis

Acquired images were analysed in Fiji/ImageJ [35] using custom-made macros. Co-localization analysis was performed per cell, where image contrast for both compared channels/measurements was enhanced (saturated = 0.35), except when using *Z*-stack images, followed by co-localization analysis with the BIOP JACoP plugin. To quantify biotinylation efficiency for enzyme modularity, masks for RNA were created, applied to raw images of streptavidin dye, and afterwards the intensity of the overlapping area was measured. To quantify the effect of crowding agents and time extension on the biotinylation radius, masks for RNA and streptavidin signals were created (Supplementary Fig. S2A, top), RNA signal area was then subtracted from the biotinylation area and divided by 2. To quantify the effect of crowding agents on biotinylation signal intensity, a mask for RNA signal was created and subsequently overlaid on the streptavidin signal. For image deconvolution, we applied the Diffraction PSF 3D plugin with adjusted parameters (index of refraction, NA, wavelength, image size, slice spacing, and number of slices), followed by usage of the parallel spectral deconvolution [36] plugin with the Tikhonov (reflexive) method and customized regularization parameters.

### Fluorescence correlation spectroscopy

WT IDG3.2 mESCs were plated on Geltrex™ hESC-Qualified coated 8-well glass-bottom IBIDI plates 1 day prior to fixation. Cells were fixed with 0.5 mg ml^−1^ DSP in 1× PBS for 40 min at room temperature, washed three times with 1× PBS and 20 mM Tris–HCl, pH 7.5, for 5 min each wash, and permeabilized with 70% ethanol overnight at 4°C. The next day, cells were rehydrated with two washes of 2× SSC, followed by three washes of 1× PBS, for 5 min each wash, and cell nuclei were stained with DAPI (1:3000 dilution) in 1× PBS for 8 min at room temperature and then rinsed twice with 1× PBS. Cells were left in different labelling solutions, sealed with parafilm, and saved at 4°C till the fluorescence correlation spectroscopy experiment. As a tracer molecule, 80 nM Alexa Fluor 647 NHS was used. We acquired the fluorescence correlation spectroscopy (FCS) data with the above-described Abberior Instruments microscope steered with our automated Python script, which moved across wells with different samples and determined regions for the FCS recordings within a nucleus (based on the DAPI signal), in the cytoplasm (i.e. in the immediate vicinity of the nucleus) and outside the cells. Within each chosen spot, three FCS measurements were taken, recording the fluorescence fluctuations for 30 s with sampling step 2 μs. The excitation power of the pulsed 640 nm laser was set to 5–10 μW at the sample plane. For each sample, at least 50 measurements for each location were performed; i.e. for the six wells, almost 1000 FCS recordings were autonomously acquired over the course of ~10 h.

**Figure 2.**
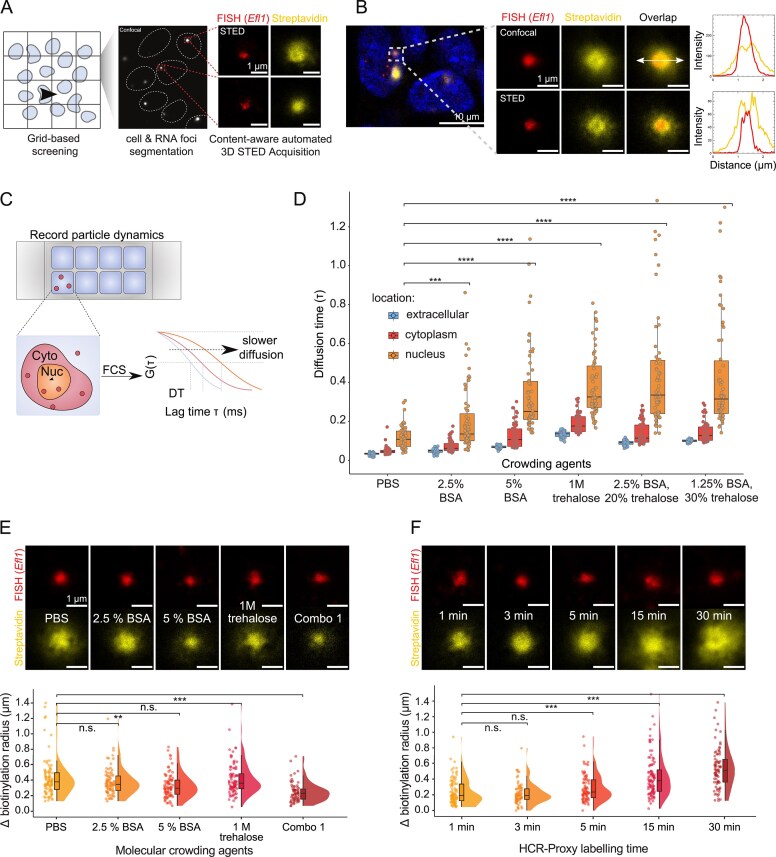
Spatially-restricted enhanced proximity labelling workflow limits the diffusion of activated biotin radicals. (**A**) Schematic workflow of the STED image acquisition platform. In each coarsely sampled confocal image of the tiled panorama scan, nuclei and RNA foci (*Efl1*) were automatically identified for subsequent super-resolution STED imaging. (**B**) STED microscopy reveals non-specific biotinylation. STED micrographs and intensity plots of the biotinylation signal (yellow, Alexa Fluor 647-conjugated streptavidin) beyond the RNA FISH (*Efl1*) boundary (red, DyLight594-conjugated anti-DIG). Reduced intensity of the biotinylation signal in the condensate’s core is barely detected with confocal microscopy. (**C**) Schematic workflow of the FCS experiment to infer the particle diffusion dynamics (red dots present the tracer molecule Alexa Fluor 647) sampled in the nucleus, cytoplasm, and extracellular compartment. Autocorrelation curves shifted towards longer lag times indicate slower diffusion. Characteristic diffusion times (DTs) shown in (D) were obtained by fitting experimental autocorrelation curves with a 3D diffusion model. (**D**) Quantification of diffusion time for the Alexa Fluor 647 fluorophore from the FCS experiment within different cellular compartments for different conditions. Average number of measurements per condition, depicted as dots: extracellular = 30, cytoplasm = 60, nucleus = 60 (two-sided Student’s *t*-test; ***P < 0.001, **P < 0.01, *P < 0.05). (**E**) Effect of molecular crowding agents on the biotinylation specificity. Top: HCR-Proxy IF micrographs against the *Efl1* intronic transcript, where proximity labeling was performed with PBS, 2.5% BSA, 5% BSA, 40% trehalose, and a combination of BSA and trehalose (2.5% BSA and 20% trehalose). Bottom: quantification of the difference (delta) in radius size between biotinylation (yellow, Alexa Fluor 647-conjugated streptavidin) and RNA FISH (*Efl1*) signal (red, DyLight594-conjugated anti-DIG). Approximately 90 images per condition were acquired, depicted as dots (two-sided Student’s *t*-test; ***P < 0.001, **P < 0.01, *P < 0.05). (**F**) Effect of labelling time extension on the biotinylation specificity. Top: HCR-Proxy IF micrographs of *Efl1* intronic transcript, where proximity labeling was performed under five different labelling times: 1, 3, 5, 15, and 30 min, respectively. For this experiment, labelling solution Combination 1 was utilized. Bottom: quantification of the difference (delta) in radius size between biotinylation (yellow, Alexa Fluor 647-conjugated streptavidin) and RNA FISH (*Efl1*) signal (red, DyLight594-conjugated anti-DIG). Approximately 90 images per condition were acquired, depicted as dots (two-sided Student’s *t-*test; ***P < 0.001, **P < 0.01, *P < 0.05).

The time traces were autocorrelated and analysed using a 3D diffusion model with a triplet component with the open source FoCuS-scan software (https://github.com/dwaithe/FCS_scanning_correlator) [37]. The fitted diffusion transit times are plotted in Fig. 2C and D.

### Mass spectrometry data analysis

**Figure 3.**
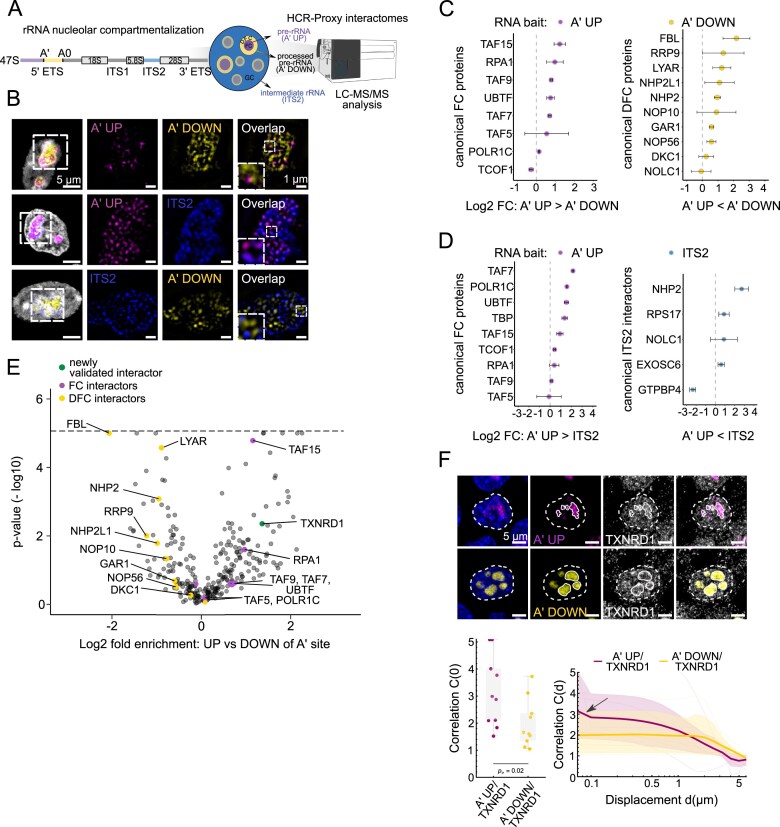
Spatially resolved rRNA-proximal proteomes within subnucleolar compartments. (**A**) Schematics of probes designed for HCR-Proxy MS to spatially resolve pre-rRNA proteomes within multiphased nucleoli. (**B**) HCR-FISH deconvolved STED micrographs of probes targeting distinct regions of the nascent rRNA transcript (magenta, A′ UP; yellow, A′ DOWN; blue, ITS2). (**C** and **D**) Dot plots displaying enriched subnucleolar HCR-Proxy components, demonstrating the ability of HCR-Proxy MS to spatially resolve multiphased nucleolar interactomes between (C) A′ UP and A′ DOWN, and (D) A′ UP and the TS2 region, respectively. Error bars present the SDs. (**E**) Volcano plot of enriched interactors specific for A′ UP region (magenta), its newly validated interactor (green) and A′ DOWN region (yellow). The cut-off for identification of *bona fide* interactors was set on LASSO regularization-based log2 fold-change > 1 and associated −log10(*P*-value) > 1. For visualization purposes, −log10(*P*) values (*y*-axis) were thresholded at 5 (dashed line). (**F**) HCR-FISH IF of the newly validated A′ UP interactor. RNA FISH (magenta, A′ UP; yellow, A′ DOWN) and newly validated nucleolar protein localized to FC (grey, TXNRD1). Quantification of the signal co-localization between RNA target (magenta, A′ UP; yellow, A′ DOWN) and candidate protein TXNRD1 with the correlation analysis; direct image correlation at zero displacement (i.e. overlap, left), and displacement-dependent correlation (right; C > 1 indicates above-random co-localization; see the Materials and methods for details). Each dot or thin curve represents one of the ~10 images per condition acquired, the bands represent 80% confidence intervals, and thick curves the mean values (one-way Mann–Whitney test; ***P < 0.001, **P < 0.01, *P < 0.05).

The MS raw files were searched using MaxQuant 1.6.3.3 against the mouse (*Mus musculus*) Proteom Fasta file extracted from UniProt (2023). Default MaxQuant settings were used with the exception of enabling label-free quantification (LFQ), match between runs, and re-quantify options. MaxQuant output was analysed using R (version 4.3.0). Log2-transformed LFQ values were used for differential protein abundance analysis. Proteins only identified by site, reverse matches, potential contaminants, and protein quantified in <3 (Supplementary Fig. S3B, C) or all (Fig. 3C, D, E) replicates of any condition were removed from the analysis. Missing values were imputed by sampling from the normal distribution derived from valid measurements, which was down-shifted by 1.8 standard deviations (SDs) and squeezed by a factor of 0.3.

The dataset consisting of targeting pre-rRNA and non-coding RNAs (ncRNAs) *Malat1* and *Norad*, along with the intronic *Efl1* sequence, was analysed in the following manner: protein abundances from pre-rRNA samples were compared with a union of those from *Malat1, Norad*, and *Elf1* using two-sided Student’s *t*-test. Thus, obtained *P*-values were false discovery rate (FDR)-corrected. The following criteria were used for considering proteins as significant nucleolar proteins: enrichment in pre-rRNA over the other targets > 1 (log2 fold change) and FDR-adjusted *P* < 0.05.

The dataset employing probes against A′ UP, A’ DOWN, and internal transcribed spacer 2 (ITS2) regions also included different labelling times and was analysed using linear modelling based on LASSO statistics. Only protein groups quantified through at least two distinct peptides were used for the analysis.

The following experiment design was used for differential protein abundance analysis: log2(LFQ) ∼ labtime + labtime:bait, where labtime refers to labelling time [either 1, 3 (only measured for A′ DOWN), 5, or 15 min], and bait refers to HCR-Proxy targets (either A′ UP, A′ DOWN, or ITS2). The following effects were thus estimated: the effect of labelling time across all targets relative to 1 min labelling; and the labelling time-dependent enrichment of protein interactors for A′ DOWN and ITS2 relative to A′ UP. The estimation of LASSO model parameters was performed using the R package glmnet [38, 39] (version 4.0.2) with thresh = 1e‐28, maxit = 1e7, and nfolds = 11. The exact model coefficients and lambda value at cross‐validation minimum (lambda.min) were extracted and used for *P*‐value estimation by fixed‐lambda LASSO inference using the R package selectiveInference [40], version 1.2.5. Default parameters were used with the following modifications: tol.beta = 0.025, alpha = 0.1, tailarea_rtol = 0.1, tol.kkt = 0.1, and bits = 100. The bits parameter was increased to 300 or 500 if the convergence was not reached. The sigma was explicitly estimated using function estimateSigma from the same package. No multiple hypothesis *P*‐value correction was performed since that is facilitated by the unbiased selection of optimum lambda—and thereby the model—for each analysed protein separately. For defining significant proteins for A′ DOWN and ITS2 regions, we considered proteins with modelled effect > 0 and associated *P*-value < 0.05, while for the A′ UP region we considered proteins with modelled effect < 0 and associated *P*-value < 0.05.

### Gene Ontology term enrichment analysis

Enrichment of nucleolar‐localized proteins was assessed against a background of all UniProt‐annotated nuclear proteins using the STRING database (v12.0) [41], considering only the overlap as target. The top 10 enriched Gene Ontology cellular component terms, ranked by the lowest FDR, were selected and mapped onto the GO‐basic ontology graph to construct a directed acyclic subgraph with their ancestral nodes. Non‐enriched parent terms were pruned to streamline the visualization, with node size scaled to the observed protein count and node colour graded by −log10(FDR).

### Modelling protein compartment identity

To identify molecular features that distinguish HCR-Proxy-defined A′ DOWN and A′ UP proteins from generic nuclear proteins, we assembled three protein sets comprising 84 A′ DOWN proteins, 118 A′ UP proteins, and 5867 nuclear proteins. To increase computational efficiency, the nuclear proteome was filtered to 5706 proteins with lengths of ≤2000 amino acids. The nuclear reference set was extracted from UniProt [42] using the filters taxonomy_id:10 090, go:0 005 634, and reviewed:true, with all proteins assigned to the A′ UP, A′ DOWN, and ITS2 proteomes excluded. Using these datasets, we developed three supervised learning frameworks, each based on a distinct protein feature representation, to classify proteins by compartment identity. Model performance was evaluated using the macro-averaged area under the receiver operating characteristic (ROC curve) (AUC) in a one versus rest setting (AUROC).

The first framework operated directly on full-length amino acid sequences. Sequences were one-hot encoded, padded to the length of the longest protein, and processed using a deep convolutional neural network. The architecture comprised five convolutional blocks, each with 128 filters and a kernel size of 10, followed by max pooling with pool size 2. A global average pooling layer aggregated positional features, which were then passed to a 64-unit fully connected layer with dropout before final softmax classification. The model was trained using the Adam optimizer with a learning rate of 1 × 10^−4^ and categorical cross-entropy loss. Early stopping was applied based on validation loss with a patience of 25 epochs and a maximum of 1000 training epochs.

In the second framework, proteins were represented using contextualized sequence embeddings derived from the ESM2 650M, 3B and 15B protein language model [43], with 650M achieving the best AUROC performance. Embeddings were extracted from the 33rd hidden layer and averaged across residues to generate a fixed-length, 1 280-dimensional vector per protein. These vectors were used as input to a fully connected neural network consisting of two hidden layers of 128 and 64 units, respectively, each followed by batch normalization and ReLU activation, and a final softmax output layer. The optimization strategy, loss function, and training protocol were identical to those used in the first framework.

The third framework used curated protein-level features rather than raw sequence representations. In total, 3481 features were computed using the localCIDER [44] and NARDINI [45] toolkits, together with annotated UniProt protein features [42]. localCIDER features captured physicochemical properties of protein sequences, including amino acid composition, fraction of charged residues, net charge per residue, isoelectric point (pI), molecular weight, and mean hydropathy. NARDINI quantified non-random clustering of residue classes using *z*-scores derived from 100 000 composition-matched sequence scramblings, capturing diagonal and off-diagonal blockiness for eight residue classes: polar, hydrophobic, positively charged, negatively charged, aromatic, alanine, proline, and glycine. UniProt annotations were incorporated to measure the fraction of each protein covered by each domain, compositional bias regions, and annotated disordered segments. All features were MinMax-normalized prior to modelling. Classification was performed using a fully connected neural network with the same architecture as the second framework. To reduce model complexity while retaining informative features, we first trained a logistic regression model on each feature individually and retained only those achieving an AUROC > 0.5, resulting in a reduced feature set of 713 features for the final model.

All three models were trained using stratified 3-fold cross-validation, with class weighting applied to the loss function to account for substantial class imbalance. For the deep learning models, 10 replicate runs were trained per fold, and the best-performing model was selected based on validation AUROC. As the feature-based model achieved the highest predictive performance overall, we employed integrated gradients to quantify individual feature importance. Integrated gradient attribution scores were computed by averaging gradients across 50 interpolation steps between a zero baseline and the true input. To ensure that attributions reflected genuine class-discriminative contributions rather than class imbalance, integrated gradients were weighted using class-balanced sample weights derived from one-hot labels for the final averaging.

## Results

### HCR-Proxy: modular approach for targeted *in situ* proximity labelling through amplified RNA recognition

Several RNA *in situ* hybridization techniques have been developed for visualization of subcellular localization of individual RNA transcripts [46, 47]. Among them, the *in situ* HCR offers an unparalleled specificity, sensitivity and high signal-to-noise ratio [30, 48]. Unlike traditional *in situ* hybridization approaches, where signal intensity depends on the number of directly labelled probes hybridized to the target, the HCR approach requires programmable oligonucleotide probes to hybridize in pairs to adjacent regions of the selected RNA. Specifically bound probe pairs trigger the signal amplification by the self-assembling chain reaction of fluorophore-labelled hairpins. To date, this method has been applied for fluorescence *in situ* hybridisation (FISH) [29, 48, 49].

Building on the strengths of HCR-FISH, we sought to extend the advantages of HCR signal amplification for a PL approach to precisely identify biomolecules interacting with or in close proximity to a specific RNA target. Therefore, we developed HCR-Proxy, a hybrid *in situ* proximity labelling workflow composed of signal amplification (HCR) [30, 46] and biotinylation step (Proxy) (Fig. 1A).

First, we developed metastable DIG-labelled hairpins (DIG hairpins) that specifically bind only the fully assembled initiator sequence to trigger the growth of a tethered DIG amplification polymer (Fig. 1A). All non-specifically bound probes and hairpins stay kinetically trapped and therefore do not trigger amplification. The amplified number of DIG molecules at the target RNA serves as a binding pad for a recombinant PL enzyme, triggering PL of adjacent biomolecules *in situ* while the unbound enzyme is washed off. To preserve biomolecular interactions, we chemically fix cells with the cell-permeable and reversible cross-linker DSP [50, 51], an amine group-specific alternative to paraformaldehyde, which can distort interactions and thus change the condensate’s composition [52]. Upon permeabilization, cells are transferred into tubes, where subsequent HCR-Proxy steps (see the Materials and methods) are streamlined, making the protocol more user-friendly and cost-efficient (Fig. 1A). Finally, biotinylated proteins are enriched by magnetic streptavidin beads under denaturing conditions followed by identification with MS (HCR-Proxy MS).

To validate the HCR-Proxy methodology, we targeted *Malat1* (Supplementry Table S1), a hallmark ncRNA of nuclear speckles [53], in mESCs. As expected, the HCR-FISH signal co-localized with the well-known nuclear speckle marker, SC35 (Fig. 1B) [7]. To assess the method’s biotinylation efficiency, we further performed PL and stained for fluorescently labelled target RNA and surrounding biotinylated biomolecules. Pearson correlation analysis (PCC) revealed strong spatial co-localization with a high degree of signal overlap (Fig. 1C). To evaluate the specificity of the method, we also conducted control experiments by omitting either the hairpin amplifiers or the probe pairs. Both conditions resulted in the absence of FISH and streptavidin signals, demonstrating the requirement for complementarity effect between probes and amplifiers to achieve biotinylation (Fig. 1C). This demonstrates that HCR-Proxy can accurately proximity label RNA in its native cellular environment.

To evaluate the enhanced biotinylation efficiency achieved by signal amplification, we designed two sets of probes targeting the intronic region of *Efl1* mRNA (Supplementary Table S1), localized to two nuclear puncta. First we aimed to identify the PL enzyme that would provide the strongest and most specific biotinylation on low abundant RNA sites. To achieve this, we purified recombinant enzymes APEX2 [25] and TurboID [18], fused to an optimized binder selective for DIG (DIG-binding domain) [54] (Supplementary Fig. S1). We observed an ~5.5-fold higher biotinylation efficiency with APEX2–DIG, yielding a high biotinylation signal-to-noise ratio while maintaining specific co-localization with the target RNA (Fig. 1D). These results demonstrate that HCR-Proxy methodology can be used in conjunction with a versatile range of PL enzymes and indicates that HCR-Proxy efficiently biotinylates even the proximity of low abundant nascent RNAs using only two sets of probes (Fig. 1E).

### Enhancing spatial specificity through biophysical tuning of labelling conditions

Next, we implemented automated content-aware STED super-resolution microscopy [55] to measure the biotinylation diameter *in situ* and to screen for conditions to improve sensitivity and resolution of the proximity labeling reaction. A fully automated STED image acquisition platform with data-adaptive imaging, similar to implementation reported in [56] (see the Materials and methods for details), provided an efficient capture of high-resolution biotinylation signal distributions, minimizing operator-induced variability and enhancing data reliability and reproducibility (Fig. 2A). For assessing labelling specificity, we chose the *Efl1* transcript as the study RNA. Its foci-like structures enabled straightforward image acquisition and subsequent signal quantification. Super-resolution imaging revealed that biotinylation staining extended beyond the boundaries of *Efl1* RNA localization, spreading within a micrometre range (Fig. 2B), consistent with previous reports of other PL approaches [25]. Surprisingly, rather than the intensity peak overlapping with the RNA FISH signal, we detected a reduced biotinylation signal in the condensate core compared with its periphery, resulting in a ‘halo labelling effect’ and reduced labelling specificity of cellular microenvironments (Fig. 2B).

To overcome the challenge of non-specific biotinylation, we next aimed to spatially constrain biotin radicals by utilizing inert crowding agents that either increase the buffer viscosity (PEG [57] and various sugars [58]) or alternatively elevate the local protein concentration (BSA [58, 59]). To assess whether these crowding agents reduce the diffusion of activated biotin radicals, we performed FCS, which measures molecular diffusion dynamics based on intensity fluctuations as molecules diffuse through a defined volume of light [60]. Fixed and permeabilized cells as per the HCR-Proxy protocol were stained with DAPI and different solutions of crowding agents were applied. PBS served as a reference labelling solution. To approximate the behaviour of the biotin radical, we used Alexa Fluor 647 fluorophore and recorded the mobility of molecules within nuclei, the cytoplasm, and the extracellular compartment. Fitting autocorrelation functions with a 3D diffusion model, we obtained the diffusion times (i.e. average time for molecules to diffuse through the focal volume) for each condition (Fig. 2C), which revealed a significant effect of crowding agents on diffusion time in all three compartments. As expected [58, 59], diffusion was slower within nuclei compared with the other two compartments, regardless of labelling solution (Fig. 2D). These findings show that molecular crowding plays a crucial role in modulating diffusion dynamics and, as such, biotinylation signal. The strongest effect was observed with a combination of BSA and trehalose (Fig. 2D), leading to a significant decrease in diffusion rate. These results demonstrate that controlled modulation of diffusion dynamics can be achieved through tailored crowding conditions, providing a useful strategy for enhancing labelling specificity in PL experiments.

To further validate our findings, we performed an HCR-Proxy IF experiment using the extended list of labelling solutions. Using our automated STED screening platform, we assessed the effect of molecular crowding agents on biotinylation specificity by comparing the radius of RNA FISH and biotinylation signal (Fig. 2E; Supplementary Fig. S2A top). The most efficient confinement of the biotin radical with its preserved signal intensity was observed with a synergistic combination of BSA and trehalose at their lower concentrations (Fig. 2E; Supplementary Fig. S2B). In addition to reducing the labelling radius, crowding agents also abolished the ‘halo labelling effect’. We propose that the decrease in radical mobility not only spatially constrained radicals to the site of their production but also prevented the inactivation of PL enzyme.

Next, we investigated whether crowding agents could compensate for extension of labelling time without compromising specificity, thereby increasing biotinylation sensitivity and consequently higher downstream yield. To determine the optimal labelling time, we performed the HCR-Proxy IF experiment at five different time points and measured the biotinylation radius surrounding the targeted RNA (Supplementary Fig. S2A, bottom). We were able to extend the standard 1 min labelling time of HCR-Proxy PL to 3 or 5 min without any noticeable spread in biotinylation radius. However, extending the labelling time to 15 or 30 min led to a significant increase in biotinylation radius and reappearance of the ‘halo labelling effect’ (Fig. 2F). These results indicate that fine-tuning the biotinylation efficiency by a combination of crowding agents and labelling time extension enhances the biotinylation yield without compromising specificity, thereby limiting the *in situ* PL reaction to individual microenvironments of biomolecular condensates.

Upon technically refining labelling and crowding conditions, we next aimed to apply HCR-Proxy to characterize the proximal proteomes of individual RNA microenvironments. For this we performed HCR-Proxy LC-MS/MS (liquid chromatography coupled to tandem MS) in mESCs with probes targeting nucleolar pre-rRNA and numerous nuclear RNAs, such as the ncRNAs *Malat1* and *Norad*, along with the intronic *Efl1* sequence (Supplementary Table S1; Supplementary Fig. S3A; Fig. 1B). This allowed us to quantify levels of 1130 proteins with varying abundances across different HCR-Proxy targets. Consistent with the distinct cellular localization of the targets, Uniform Manifold Approximation and Projection Analysis for dimensionality reduction (UMAP) showed a clear separation of samples between nuclear and nucleolar targets (Supplementary Fig. S3B). We considered proteins as *bona fide* nucleolar proteins if they were significantly enriched over the remaining nuclear targets (Student’s *t*-test-based log2 fold change > 1, FDR-adjusted *P*-value < 0.05) (Supplementary Fig. S3C), thereby obtaining 602 nucleolar proteins, including numerous well-established pre-rRNA interactors (Supplementary Tables S2 and S3). Consistent with the data, GO term annotation of the pre-rRNA proximal proteome showed an enrichment for ribosomal proteins (Supplementary Fig. S3D). Furthermore, comparison with publicly available PL-based nucleolar datasets [25, 61] showed a significant enrichment of the overlapped nucleolar interactors in our HCR-Proxy dataset (Supplementary Fig. S3E, F). With this, we have shown that HCR-Proxy facilitates target-dependent enrichment of specific protein interactors of RNA and thus is capable of profiling RNA-scaffolded condensates.

### Resolving nested RNA microenvironments with HCR-Proxy

With the unprecedented specificity (Fig. 2), we postulated that HCR-Proxy for the first time paves the way towards spatially resolved proximity labeling of RNP condensates. To demonstrate the versatility of HCR-Proxy in deciphering the interactome within individual condensates, we aimed to delineate the multiphased composition of nucleoli, which consists of (at least) three subcompartments [62]. For this we targeted different regions of the nascent rRNA transcript: (i) upstream (A′ UP) or (ii) downstream (A′ DOWN) of A′ processing and (iii) spanning ITS2 region (Supplementary Table S1), postulated to be localized to the fibrillar centre (FC), the dense fibrillar component (DFC), and the granular component (GC), respectively (Fig. 3A). Using HCR-FISH staining, we observed mutually exclusive signals for all three targeting sites (RNA baits) (Fig. 3B), confirming their distinct spatial distribution within the nucleolus.

At the same time, each of them also co-localized with its known subnucleolar marker (FC (POLR1A), DFC (FBL), GC (NPM1)) (Supplementary Fig. S4A). In agreement with the nucleolar localization of all three pre-rRNA targets, HCR-Proxy MS identified distinct subnucleolar proximal proteomes (Supplementary Table S4). When comparing the HCR-Proxy interactomes for nascent pre-rRNA regions, A′ UP and A′ DOWN, we detected prominent components for rRNA transcription and established FC markers enriched in the A′ UP region. The latter enrichment includes numerous transcription factors responsible for forming the Pol I pre-initiation complex such as UBTF [9, 63, 64], TAF15, TAF9, TAF7, and TAF5 [65], alongside the POLR1C component of the RNA Pol I complex [9] and RPA1 protein that is involved in rRNA transcription regulation [66] (Fig. 3C, left). In contrast, numerous proteins involved in early stages of pre-rRNA processing, including FBL [9, 67], its interaction partner GAR1 [67], LYAR, and various components of the box C/D and H/ACA small nucleolar ribonucleoprotein (snoRNP) complexes, such as NHP2, NOP56, and NOP10 [9, 64], were enriched in the HCR-Proxy interactome specific for the A′ DOWN region (Fig. 3C, right). We were also able to distinguish the recently identified ITS2 interactors [13, 68] when compared with the A′ UP region, where we recapitulated some of the known components responsible for its processing such as RPS17, NHP2, NOLC1, and EXOSC6 (Fig. 3D).

Encouraged by the recovery of many known interactors specific to each pre-rRNA region, we next sought to investigate whether HCR-Proxy could reveal previously unreported subnucleolar components. To this end, we then selected thioredoxin reductase TXNRD1 as a candidate protein, which had not been previously characterized within the nucleolus (Fig. 3E). Its IF imaging showed a higher ratio of spatial correlation with the A′ UP bait region compared with the A′ DOWN region (Fig. 3F), aligning with obtained proteomics data (Fig. 3E). These results combined indicate that HCR-Proxy not only distinguishes interactomes from spatially restricted RNA assemblies within the same condensate, but also offers a powerful strategy for uncovering novel subnucleolar components.

### Sequence-encoded logic of condensate partitioning based on HCR-Proxy interactomes

**Figure 4.**
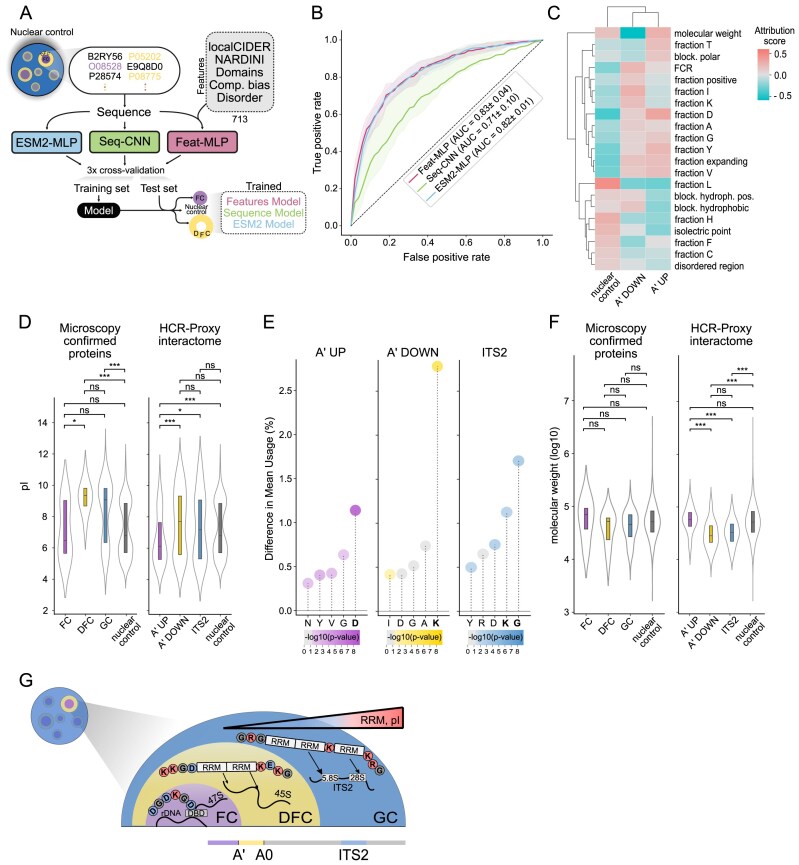
Protein sequence code distinguishes nucleolar subcompartmental interactomes obtained by HCR-Proxy. (**A**) Schematic overview of the deep learning models trained to classify the A′ UP (FC-associated) interactome, the A′ DOWN (DFC-associated) interactome, and the nuclear proteome. Protein sequences were classified using either ESM2 embeddings, a sequence-based CNN model (Seq-CNN), or 713 manually curated protein features-based model (see the Materials and methods). Models were evaluated using 3-fold cross-validation. (**B**) Receiver operating characteristic (ROC) curves showing the one-vs-rest classification performance of each model with AUROC and SD, across cross-validation folds. (**C**) Attribution calculation of protein features included in the Features-MLP model identifying the top predictive features of A′ UP, A' DOWN, and nuclear localization, with the top eight shown per class. Abbreviations: hydroph., hydrophobic; block., blockiness; FCR, fraction of charged residues. (**D**) Comparison of the pI between nucleolar proteins confirmed by microscopy and identified by HCR-Proxy MS. Number of proteins FC = 10, DFC = 13, GC = 88 [9], A′ UP = 118, A′ DOWN = 84, ITS2 = 75, remaining GO-termed nuclear = 5867 (Mann–Whitney *U*-test; ***P < 0.001, **P < 0.01, *P < 0.05). (**E**) Comparison of amino acid usage in A′ UP, A′ DOWN, and ITS2 proximal proteomes relative to the nuclear background. The five residues with the largest differences in mean usage are highlighted. Dot colour reflects statistical significance (Welch’s *t*-test: *P < 0.05, **P < 0.01, ***P < 0.001). (**F**) Comparison of molecular weights between nucleolar proteins confirmed by microscopy and identified by HCR-Proxy MS. Number of proteins FC = 10, DFC = 13, GC = 88 [9], A′ UP = 118, A′ DOWN = 84, ITS2 = 75, remaining GO-termed nuclear = 5867 (Mann–Whitney U-test; ***P < 0.001, **P < 0.01, *P < 0.05). (**G**) Graphical summary of uncovered protein features across subnucleolar compartments.

We next postulated that with the expansion of the pool of identified proteins for each compartment, we could fully leverage deep learning (DL) modelling to further refine our understanding of the sequence-encoded determinants governing protein partitioning across nucleolar subcompartments. To achieve optimal performance and interpretability, we benchmarked three DL frameworks under stratified 3-fold cross-validation (Fig. 4A) for classifying the A′ UP or A′ DOWN region-specific interactomes against the nuclear protein control (see the Materials and methods).

The first model was a convolutional neural network (Sequence-CNN) trained on full-length amino acid sequences and designed to recognize motif patterns predictive of class membership. The second model (ESM2-MLP) leveraged embeddings derived from the transformer-based protein language model ESM2 [43] capturing global sequence context and higher-order amino acid interactions. The third model (Feature-MLP) relied on 713 curated features (Fig. 4A), including sequence-derived properties calculated using localCIDER [44], NARDINI [45], and annotated protein characteristics obtained from UniProt [42].

Surprisingly, the Feature-MLP classifier attained a mean one-vs-rest AUROC of 0.83, outperforming the protein language model-based ESM2-MLP classifier (mean AUROC = 0.82) and the Sequence-CNN (mean AUROC = 0.71) (Fig. 4B; Supplementry Fig. S4B). Attribution analysis revealed that HCR-Proxy-enriched proteins in the A′ UP interactome (associated with FC) are characterized by greater molecular weight and elevated acidic residue content, together with a lower pI (Fig. 4C). In contrast, HCR-Proxy-enriched proteins in the A′ DOWN interactome (associated with DFC) exhibit a higher fraction of charged, predominantly basic residues, with blocky hydrophobic residue patterning. Both A′ UP and A′ DOWN interactomes exhibit a lower fraction of leucine residues compared with the nuclear control (Fig. 4C).

To examine the most informative features in greater detail, we directly compared the pI of all HCR-Proxy nucleolar targets with microscopy-confirmed proteins corresponding to specific nucleolar subcompartments [9] (FC, DFC, and GC) and with the remaining nuclear proteome. Consistent with the Feature-MLP model’s identification of increased acidity, both datasets show that the A′ UP cohort has a significantly lower median pI than the A′ DOWN interactome (Fig. 4D). Per-residue enrichment analysis of the HCR-Proxy interactome showed that aspartate (D) frequencies were markedly elevated in A′ UP proteins, whereas lysine (K) predominated in the A′ DOWN and ITS2 proteomes, with leucine (L) strongly depleted across all nucleolar subcompartments (Fig. 4E; Supplementary Fig. S4C).

These model-derived features of HCR-Proxy interactomes are strongly supported by experimental dissection of protein sequences from canonical nucleolar proteins harbouring acidic D/E tracts and lysine-rich blocks [64]. The prominence of blocky acidic residue tracts among FC-enriched proteins (Supplementary Fig. S4C, D) mirrors the identification of D/E tracts as determinants of FC localization and lower local pH. Similarly, basic-residue block regions characteristic of DFC scaffolds were validated to be significantly enriched in the HCR-Proxy A′ DOWN interactome (Supplementry Fig. S4D).

Next, we compared molecular weights among HCR-Proxy targets and microscopy-confirmed proteins, and found that A′ DOWN and ITS2 proteins had markedly lower molecular weights than A′ UP and other nuclear proteins (Fig. 4F). A similar trend was observed for microscopy-confirmed proteins, although this difference did not reach statistical significance. Consistent with the established role of RNA-recognition motif (RRM) domains in nucleolar organization [69] and their identification as an informative feature in our model (Supplementry Fig. S4E), we further analysed RRM domain content across subnucleolar proteomes. RRM abundance increased progressively from A′ UP to A′ DOWN and ITS2 (Supplementry Fig. S4F), consistent with the role of DFC/GC proteins in pre-rRNA processing [64]. This indicates that A′ DOWN proteins tend to be smaller, more basic and enriched in RNA-binding capacity, including basic amino acid blocks important for RNA interaction and processing [70].

In conclusion, the rich, compartment-specific proteomic data enabled by HCR-Proxy allowed us to further uncover the molecular code underlying nucleolar organization. This data-driven insight not only identifies distinct sequence features driving spatial partitioning, but also provides support for the pH gradient model [64], in which larger, acidic FC proteins scaffold to the fibrillar centre, while smaller, basic, RRM-enriched proteins preferentially localize to the surrounding dense fibrillar and granular components to orchestrate dynamic rRNA processing (Fig. 4G).

## Discussion

Biomolecular condensates have emerged as critical hubs of cellular organization, with compartmentalization often orchestrated through RNA-mediated multivalent interactions. While considerable progress has been made in cataloguing the global composition of various condensates [71], the spatial architecture of condensate patterning or proteomic mapping within nested condensates—such as the nucleolus—have been constrained by limitations in both resolution and sensitivity of RNA-centric proteomics approaches.

To address this challenge, we developed HCR-Proxy, a proximity labeling method that leverages an HCR for both signal amplification and spatial confinement of biotinylation, thereby enabling subcompartmental characterization of RNA-proximal proteomes *in situ*. By designing DIG-labelled HCR hairpins as localized docking platforms for recombinant DIG-binding enzymes, HCR-Proxy introduces a modular and plug-and-play architecture: any recombinant enzyme fused to a DIG-binding domain (such as APEX2 or TurboID in this work) can be readily recruited to the RNA target site. This modularity allows broad adaptability across different labelling chemistries and also circumvents the constraints of conventional genetically encoded PL systems, allowing for seamless application in primary cells and complex systems where genome editing may not be feasible.

One of the further innovations in our workflow is the confinement of labelling reactions to submicroscale environments through diffusion tuning by employing macromolecular crowding agents. When combined with automated content-aware 3D STED imaging to identify optimal buffer viscosity, this approach enabled unbiased, high-resolution mapping of labelling radii and supported the development of HCR-Proxy as a biophysically tunable system for high-specificity labelling within subcompartment RNA-defined niches. Despite the diffusion confinement of labelling reactions, efficiency of biotinylation with HCR-Proxy does not require a high number of hybridization probes, but is rather the result of signal amplification achieved by DIG-labelled hairpins at the site of the targeted RNA. This enabled us to use as little as two probe sets to achieve biotinylation of proximal proteomes, even for intronic RNA targets (Fig. 1E).

Using this framework, we demonstrate that HCR-Proxy can resolve subcompartmental microenvironments within a single condensate. As a hallmark multilayered condensate, we used the nucleolus and targeted distinct regions of pre-rRNA nascent transcript, each representing one of the three nucleolar sublayers. This enabled molecular dissection of spatial proteome sublayers within a single condensate, not only revealing known markers but also uncovering novel nucleolar proteins. In this way, we expanded the repertoire of identified proteins for each subcompartment and integrated DL classifiers trained on amino acid sequences, biophysical features, and domain architectures to provide a functional link between sequence-encoded features and localization to distinct rRNA-defined subdomains. Consistent with recent findings that subcellular localization is governed by distributed ‘protein codes’ [72], our findings also reinforce the biophysical model of pH gradients within the nucleolus [64], where acidic intrinsically disordered regions containing D/E tracts scaffold the FC, creating a microenvironment distinct from the lysine-rich, RRM-rich proteins of the DFC and GC. Next, our deep learning analysis also revealed that simple, interpretable features such as molecular weight, net charge and domain content can outperform transformer-derived embeddings in classifying subnucleolar localization. This suggests that phase behaviour and spatial partitioning are deeply encoded in primary protein sequence, and this predictive logic extends prior findings from engineered systems to endogenous interactomes.

### Limitations of the study

Our study presents a novel technology with high application capacity for deciphering the proximal proteomes of spatially restricted RNA-scaffolded compartments. However, the application of PL methods is currently limited to RNAs that form condensate-like structures, while the interactomes of dispersed RNAs have not yet been elucidated and may require further technical refinements of the method. In addition, the subcompartmental resolution of RNA-scaffolded proteomes in our study was based on nucleolar RNA, which is relatively abundant, so further labeling optimizations may be required for other less abundant RNA-scaffolded condensates. Furthermore, a potential limitation of most RNA–protein mapping methods is the acquisition of averaged interactome snapshots, which may miss potential nuances in the condensate’s composition.

## Supplementary Material

gkag086_Supplemental_Files

## Data Availability

Newly produced data were deposited in ProteomeXchange with identifier PXD064165. The code and notebooks to analyse the data and to produce the figures in this work are available at: https://github.com/ModicLab/HCR_proxy. The code used for HCR-Proxy image acquisition is deposited at Zenodo (10.5281/zenodo.15489876) as are acquired microscopy images (10.5281/zenodo.15479056).
