# Supplementary figures and images for: HCR-Proxy resolves site-specific proximal RNA microenvironments at subcompartmental resolution
Source: Nucleic Acids Res. 2026 Feb 23;54(4):gkag086. doi: 10.1093/nar/gkag086 (PMC12926915; doi:10.1093/nar/gkag086)

# Supplementary Figure 4

A

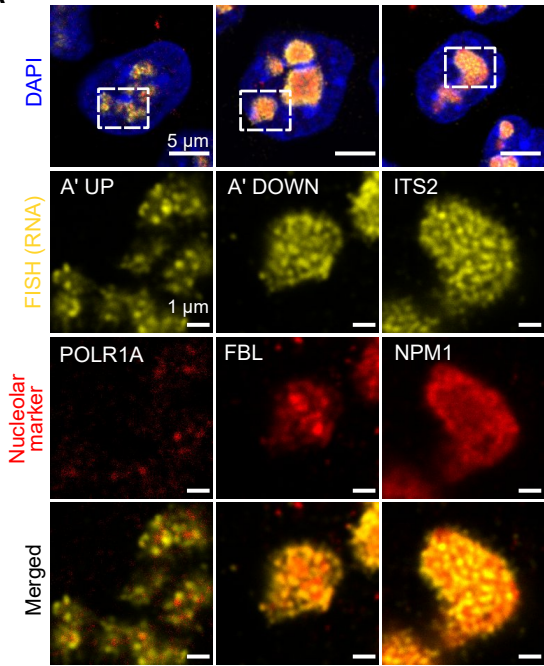

C

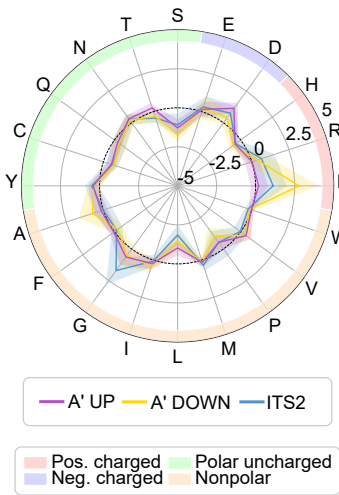

F

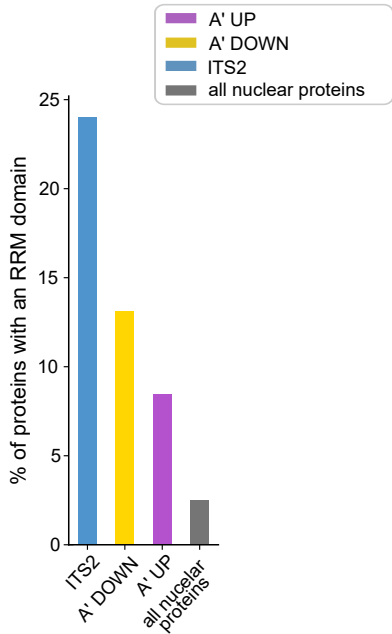

B

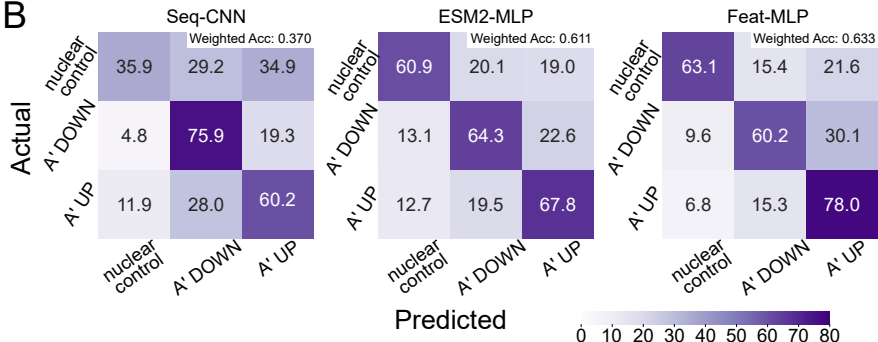

D

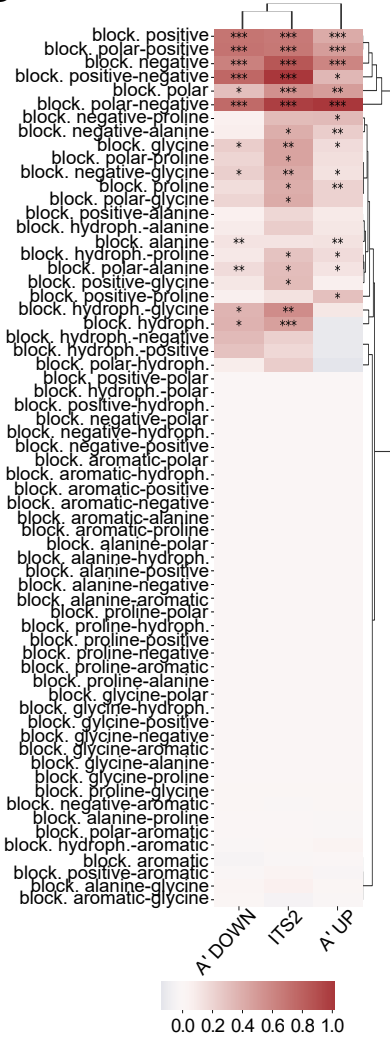

E

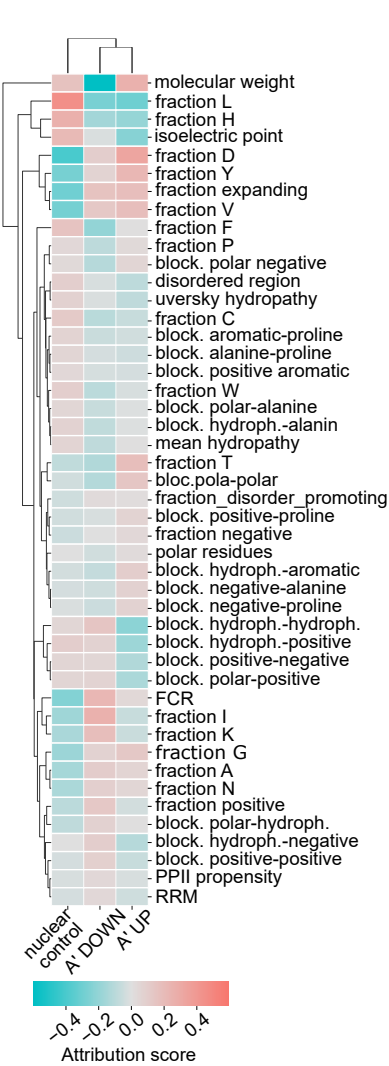

Supplement: gkag086_Supplemental_Files [file gkag086_supplemental_files.zip › Revision_Supplementary fig 4 .pdf]

# Supplementary Figure 2

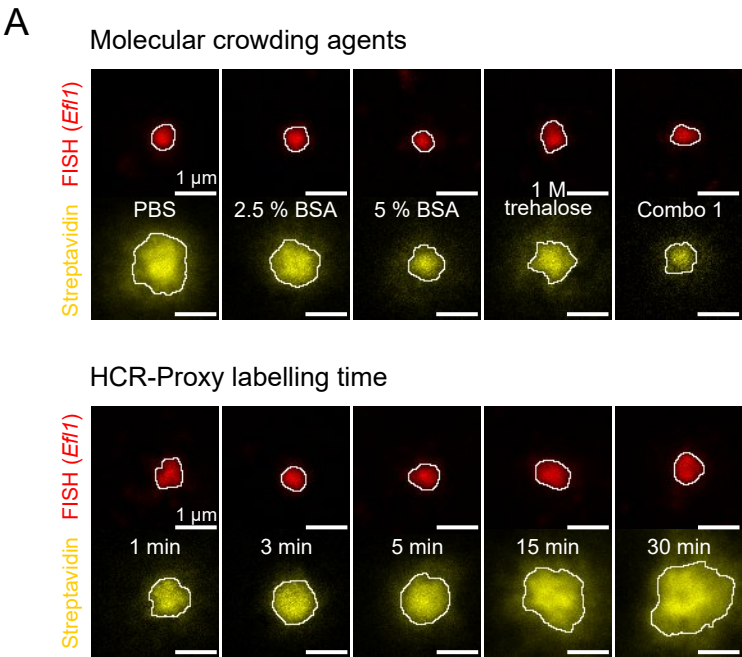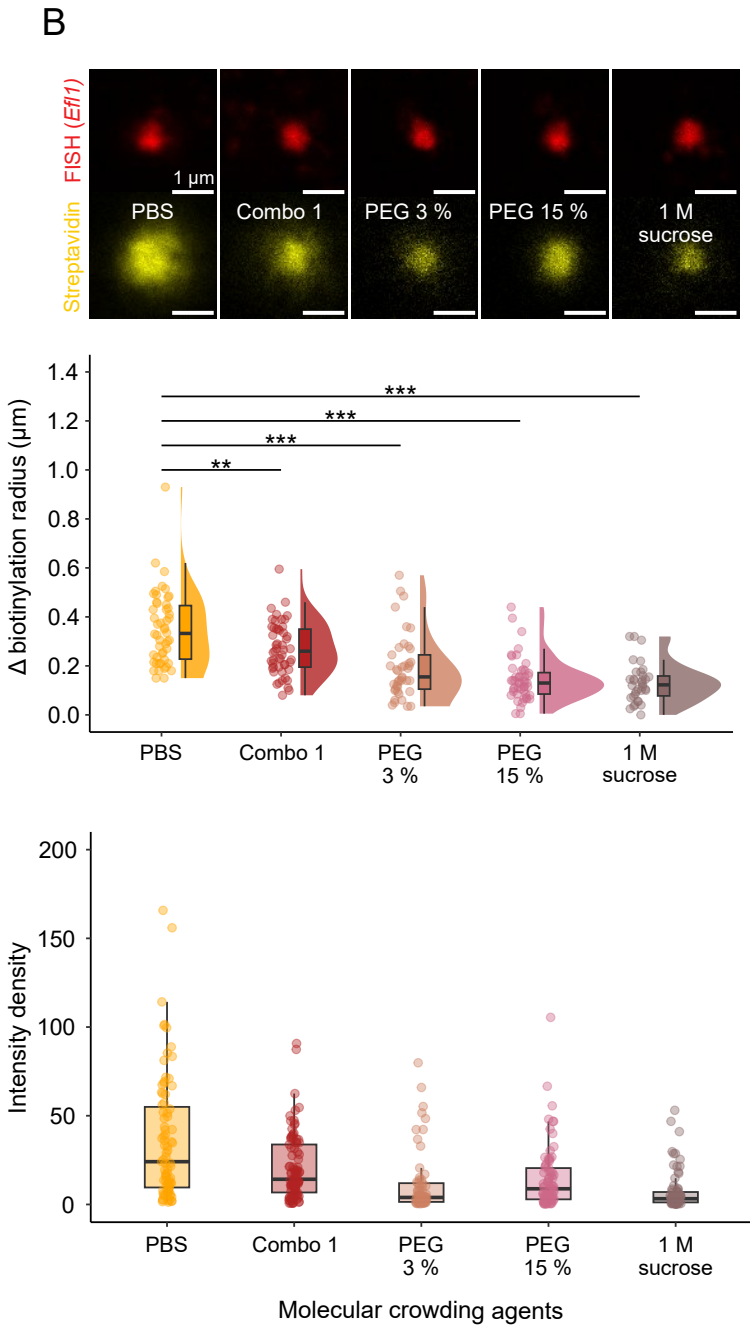

Supplement: gkag086_Supplemental_Files [file gkag086_supplemental_files.zip › Revision_Supplementary figure 2_20251217.pdf]

# Supplementary Figure 3

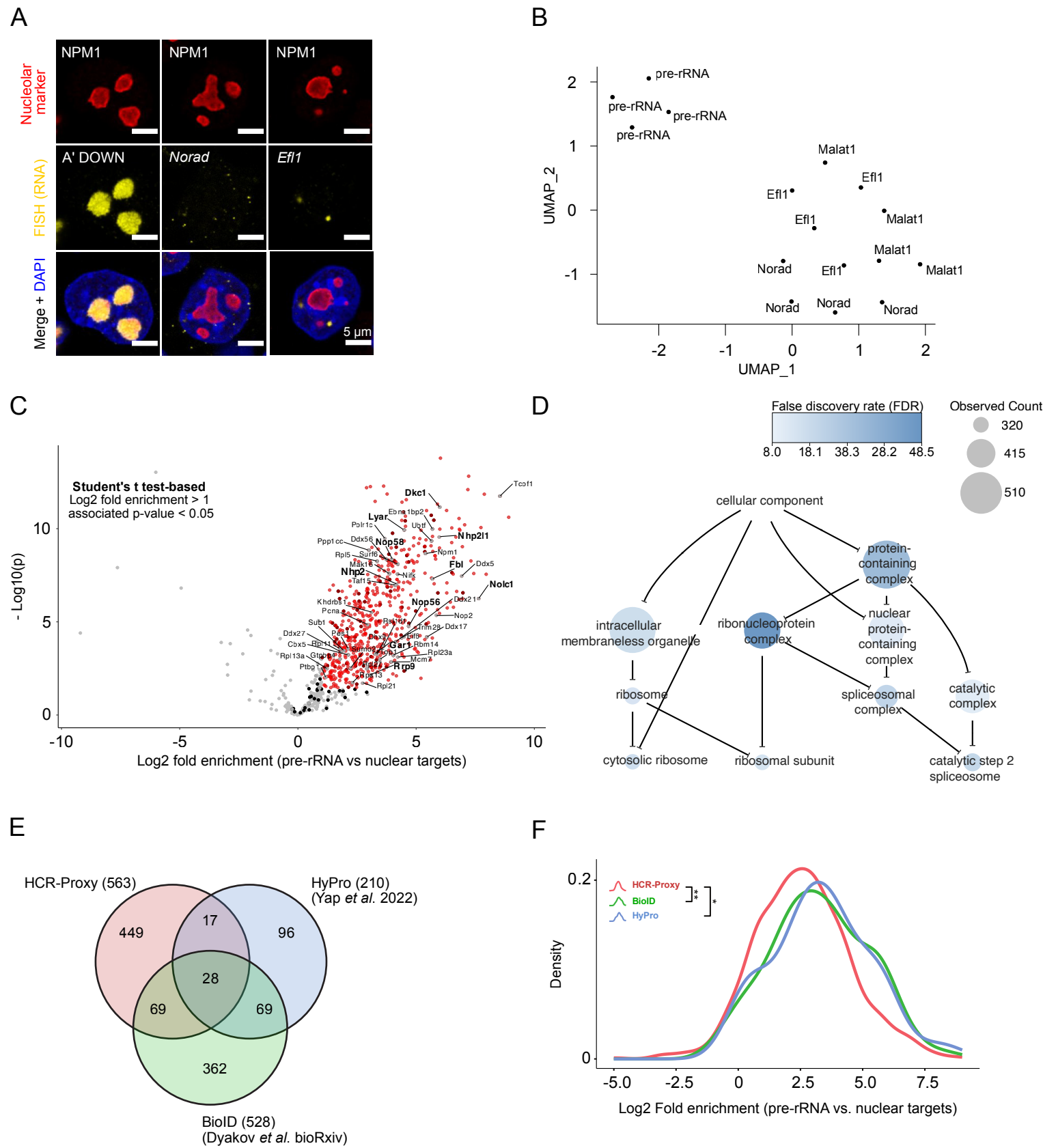

Supplement: gkag086_Supplemental_Files [file gkag086_supplemental_files.zip › Revision_Supplementary Figure 3MM.pdf]

# Supplementary Figure 1

A

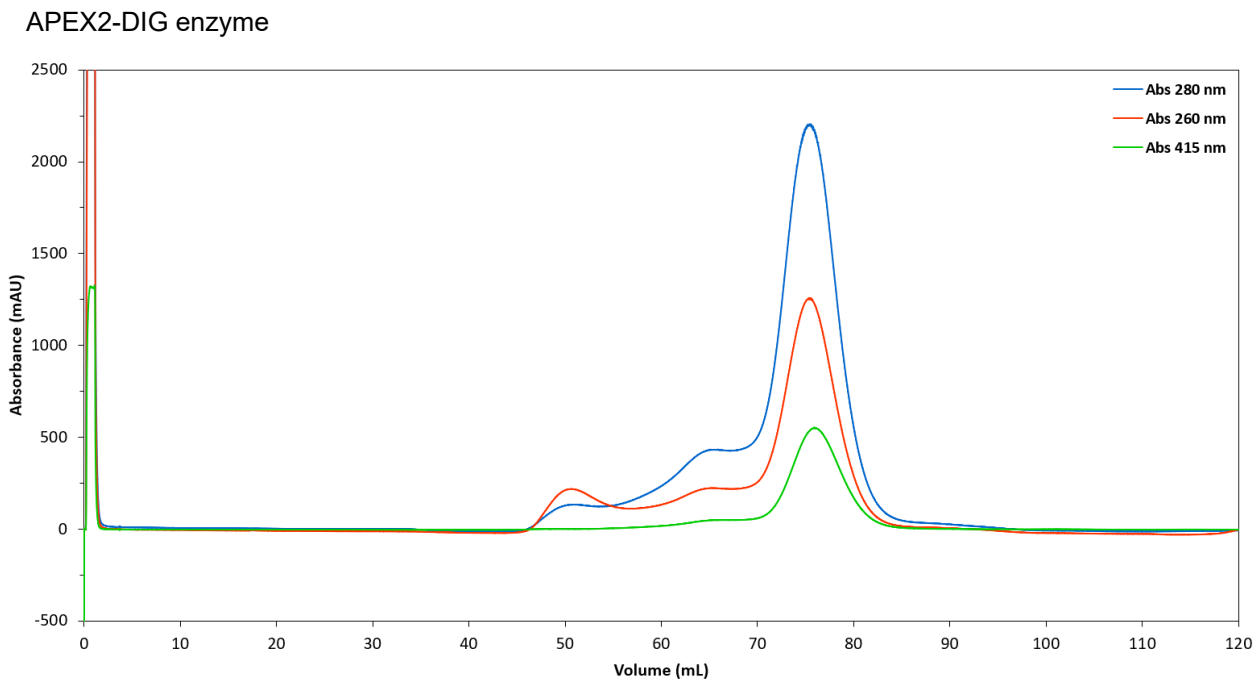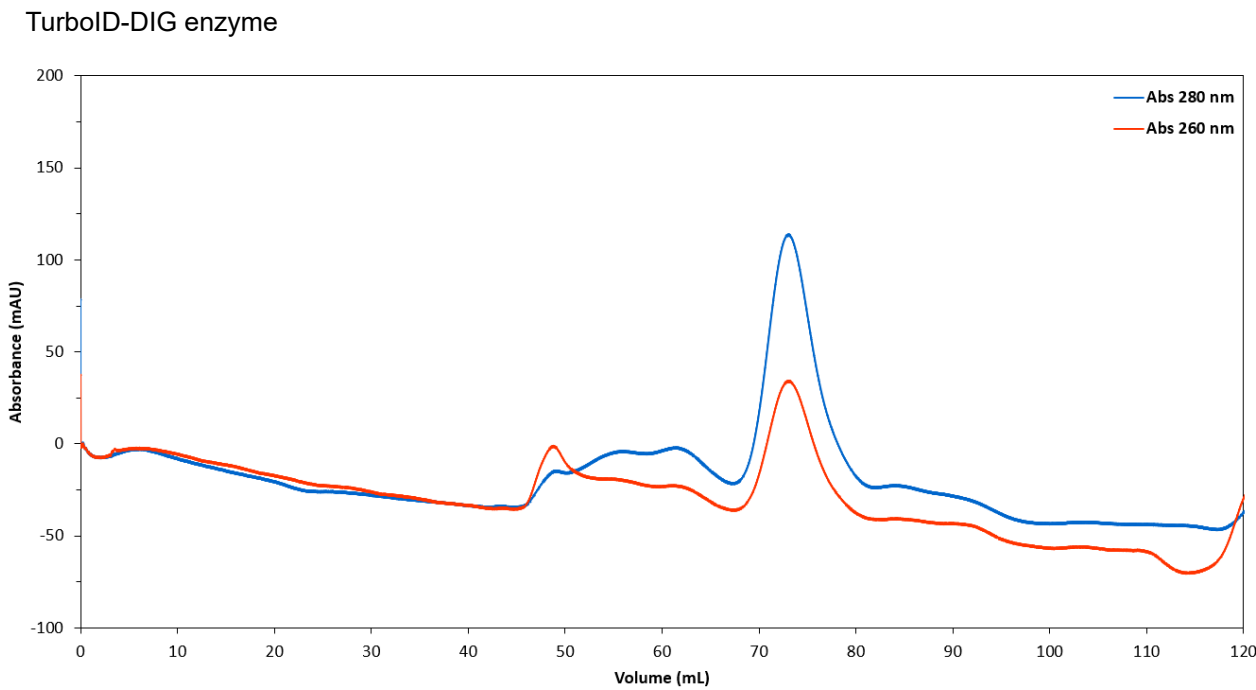

B

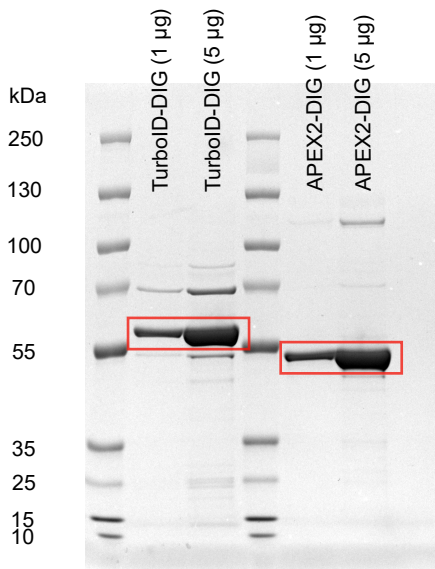

Supplement: gkag086_Supplemental_Files [file gkag086_supplemental_files.zip › Supplementary Figure 1.pdf]
