# Supplementary material for: HCR-Proxy resolves site-specific proximal RNA microenvironments at subcompartmental resolution
Source: Nucleic Acids Res. 2026 Feb 23;54(4):gkag086. doi: 10.1093/nar/gkag086 (PMC12926915; doi:10.1093/nar/gkag086)
Supplement: gkag086_Supplemental_Files [file gkag086_supplemental_files.zip › Supplementary Figures_Response to referees_Final_20260119.docx]

**Supplementary Figures**

**Supplementary figure 1: Isolation of PL enzymes TurboID-DIG and APEX2-DIG used for HCR-Proxy**

A. *Elution profiles from size exclusion chromatography for isolated PL enzymes.*
B. *SDS-PAGE analysis of purified PL protein peaks eluted from a size exclusion chromatography column. Bands within red rectangles represent isolated enzymes.*

**Supplementary figure 2: Fine tuning of labelling conditions constricts biotinylation radius**

A. *HCR-Proxy IF micrographs of Efl1 transcript with mask overlays for radius quantification of RNA FISH (red, DyLight594-conjugated anti-DIG) and biotinylation (yellow, Alexa647-conjugated streptavidin) signal. Top: molecular crowding agents experiment, bottom: HCR-Proxy labelling time experiment.*
B. *Effect of molecular crowding agents on the biotinylation specificity. Top: HCR-Proxy IF micrographs against Efl1 intronic transcript, where proximity labelling was performed with PBS, Combination of BSA and trehalose (2.5 % BSA & 20 % trehalose), 3 % PEG8000, 15 % PEG8000 and 1 M sucrose. Middle: Quantification of the difference (delta) in radius size between biotinylation (yellow, Alexa647-conjugated streptavidin) and RNA FISH (Efl1) signal (red, DyLight594-conjugated anti-DIG). Approximately 50 images per condition were acquired, depicted as dots (two sided Student’s t-test; p < 0.001 = ***, p < 0.01 = **, p < 0.05 = *). Bottom: Quantification of the intensity density signal for biotinylation (yellow, Alexa647-conjugated streptavidin) within the mask for the RNA FISH (Efl1) signal (red, DyLight594-conjugated anti-DIG). Approximately 75 images per condition were acquired, depicted as dots.*

**Supplementary figure 3: HCR-Proxy enables profiling of different RNA-scaffolded compartments within the cell**

A. *HCR-FISH micrographs of pre-rRNA (A’ DOWN), Norad and intronic Efl1 sequence (yellow, DyLight594-conjugated anti-DIG) combined with immunofluorescence detection of nucleolar marker NPM1 (red, NPM1 antibody), merged with DAPI (blue).*
B. *UMAP analysis of nucleolar (pre-rRNA) and nuclear (Norad, Efl1, Malat1) targets from HCR-Proxy MS experiment (four technical replicates per RNA target).*
C. *Volcano plot for the pre-rRNA-proximal proteome. Proteins marked in red present bona fide nucleolar proteins, meanwhile literature-known nucleolar proteins are labelled* (two-sided Student's t test-based log2 fold-change > 1, FDR-adjusted p-value < 0.05*). Known DFC proteins are labelled with enlarged text.*
D. *GO term annotation of identified bona fide nucleolar protein interactors.*
E. *Venn diagram of nucleolar-associated proteins identified by the three PL approaches: HyPro[(Yap et al. 2022)](https://paperpile.com/c/7SJlt0/eGER), TurboID[(Dyakov et al. 2024)](https://paperpile.com/c/7SJlt0/VjPV) and HCR-Proxy.*
F. *Density plot depicting fold-enrichment (log2) of indicated protein sets (all - red; overlapping with Dyakov et al. 2024-green; overlapping with Yap et al. 2022-blue) over non-nucleolar controls. Significance of difference between distributions was calculated using Two sided Kolmogorov-Smirnov test; p < 0.001 = ***, p < 0.01 = **, p < 0.05 = *).*

**Supplementary figure 4: Primary sequence-derived features enable distinction between HCR-Proxy interactomes.**

A. *HCR-FISH micrographs of RNA baits targeting sublayers of multiphased nucleolus (A’ UP, A’ DOWN and ITS2) (yellow, DyLight594-conjugated anti-DIG) combined with immunofluorescence detection of FC marker POLR1A, DFC marker FBL and GC marker NPM1 (red), merged with DAPI (blue).*
B. *Confusion matrices of the deep learning models trained to classify A′ UP (FC-associated) interactome, A′ DOWN (DFC-associated) interactome, and nuclear proteome, with a labeled class weighted average accuracy.*
C. *Radar plot showing the mean difference in amino acid usage in A′ UP, A′ DOWN, and ITS2 proximal proteomes relative to the nuclear background, with 95% confidence intervals.*
D. *Clustermap of mean NARDINI pairwise blockiness Z-scores of eight amino acid residue classes for A′ UP, A′ DOWN, and ITS2 proximal proteomes. Asterisks denote statistical significance relative to zero from a one-sample Student’s t-test (p < 0.05 = *, p < 0.01 = **, p < 0.001 = ***). Abbreviations: hydroph. - hydrophobic.*
E. *Attribution calculation of features included in the model identifying the top predictive features of A′ UP,  A ′DOWN and Nuclear localization, with top 20 shown per category. Abbreviations: hydroph. - hydrophobic, block. - blockiness, FCR - fraction of charged residues.*
F. *Analysis of annotated RRM domain presence in A′ UP, A′ DOWN, and ITS2 proximal proteomes and nuclear control, showing the percentage of proteins containing at least one RRM domain.*
